# Mechanistic Insights into the Stabilizing Role of Deep Eutectic Solvents for Nucleic Acids: An *In Silico* Analysis

**DOI:** 10.1021/acs.jpcb.5c00799

**Published:** 2025-06-03

**Authors:** David E. Hardy, Adam Albert, Jenna Lipani, Zachary J. Metott, Arsalan Mirjafari, Durgesh V. Wagle

**Affiliations:** † Department of Chemistry and Physics, 3391 Florida Gulf Coast University, 10501 FGCU Boulevard South, Fort Myers, Florida 33965, United States; ‡ Department of Chemistry, 14828 State University of New York at Oswego, Oswego, New York 13126, United States

## Abstract

The cold chain system poses a significant obstacle to equitable global access to safe and effective nucleic acid-based vaccines. There is, therefore, a critical need to eliminate or improve this vaccine coverage gap by minimizing the costly and labor-intensive cold chain process, thereby providing equitable access to these life-saving therapeutics. In this work, quantum chemical evaluation qualitatively compared the interactions of a choline chloride/trehalose-based deep eutectic solvent (DES) with model bases (adenine (A), guanine (G), cytosine (C), thiamine (T), and uracil (U)) and their base pairs AT, AU, and GC in nucleic acids. We found that unpaired purines primarily interacted with the DES through C–H···π and hydrogen bonding, leading to cage-like structures, while pyrimidines primarily engaged through hydrogen bonding. In DES/base pair complexes, the AT base pair weakened and partially lost planarity due to C–H···π interactions, while the AU base pair was disrupted by additional hydrogen bonding with Cl^–^. Conversely, the GC base pair retained its structure with strengthened hydrogen bonding in the presence of DES. Aromaticity increased in unpaired bases and base pairs AT and AU due to multiple hydrogen bonds but decreased in the DES/GC complex dominated by C–H···π interactions. Charge transfer analysis showed that purines lost electron density upon interacting with DES, while pyrimidines gained electron density. Interestingly, the GC and AT base pairs that retained pairing lost electron density to DES, whereas the AU base pair gained electron density, indicating complex interactions. The thermochemistry indicated favorable interactions of DES with unpaired bases and base pairs. However, it also suggests that once the bases are unpaired, it is energetically expensive for them to pair again.

## Graphical abstract

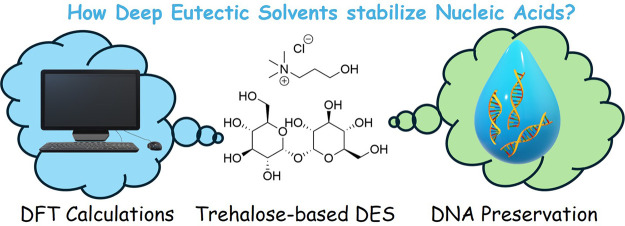

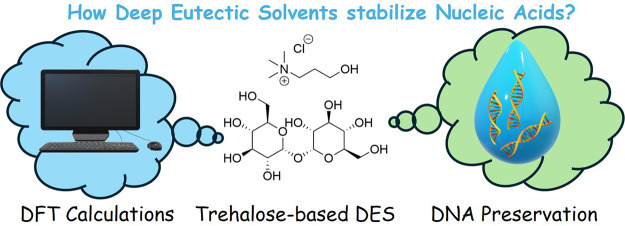

## Introduction

It was estimated that if the COVID-19 vaccines had been distributed more homogeneously worldwide during the first year, the high mortality rate could have been significantly reduced, potentially preventing 1.1–1.5 million deaths globally.^1^ mRNA vaccines, although highly effective, require stringent cold storage conditions ranging from −20 to −80 °C to prevent enzymatic denaturation and other forms of damage at ambient temperatures. This fragility presents logistical and financial challenges, especially for countries lacking adequate cold chain infrastructure necessary for vaccine transportation and storage. To improve the availability of mRNA vaccines, it is imperative to develop a storage medium capable of preserving nucleic acid-based vaccines at ambient temperature for extended time periods, thereby negating the burden of cold chain distribution. Therefore, we hypothesize that replacing common aqueous media for nucleic acid preservation with chemically tunable media, such as natural deep eutectic solvents, could significantly reduce the risk of enzymatic degradation and hydrolysis.

Deep eutectic solvents (DESs) synthesized from biocompatible materials hold the potential for long-term preservation of vaccines at ambient temperatures to eliminate the cold chain.^2^ DESs are low-melting mixtures prepared by mixing high-melting compounds (*T*_m_ > 100 °C) in specific molar ratios.^3^ Typically, a DES is prepared by mixing an ammonium salt such as choline chloride, which acts as a hydrogen bond acceptor, and a nonionic compound, which acts as a hydrogen bond donor.^3^ Most DESs are prepared from a wide variety of inexpensive, biocompatible compounds, thus making them chemically tunable, biodegradable, and environmentally friendly.^4–7^

Trehalose, a naturally occurring disaccharide, is widely utilized in the biopharmaceutical, food, and cosmetic industries due to its remarkable stabilizing and cryoprotective properties.^8^ In fact, it stands out as one of the most efficient and versatile stabilizing cosolutes, demonstrating an exceptional ability to preserve the structural and functional integrity of nucleic acids and several other biomolecules in a variety of storage conditions.^9^ The specific stereochemical arrangement of trehalose’s multiple hydroxyl groups plays a key role in the formation of unique hydrogen-bonding interactions. Compared to other sugars (e.g., sucrose), trehalose exhibits superior efficacy in maintaining cellular integrity by protecting the native three-dimensional structures of cell membranes and proteins, thereby inhibiting their denaturation, degradation, and aggregation. Additionally, trehalose possesses a higher affinity for water molecules and occupies a larger hydrated volume relative to other carbohydrates.^10^

During the past decade or so, the biological applications of DESs involving the purification and extraction of proteins and nucleic acids have slowly gained momentum. For instance, Wang et al. performed DNA extraction from E. coli using hydrophobic magnetic DESs with highly pure plasmid DNA from bacterial lysate.^11^ Similarly, a poly­(ethylene glycol)-based DES in aqueous biphasic systems was developed by Zhang et al. for RNA extraction that demonstrated back-extraction efficiency of up to 90.78% and selective partitioning of RNA and tryptophan.^12^ Belviso and co-workers demonstrated that choline-based DESs can be used to crystallize proteins. In their work, they found that the water content in DES was the key driver of protein crystallization, thus indicating that DESs can serve as suitable media for protein crystallization.^13^ In recent times, the application of DES has expanded into protein depolymerization,^14^ protein fibrillating agents,^15^ and selectively separating target proteins from mixtures through molecular imprinting of DES.^16^ More studies on testing DESs for their potential as suitable nonaqueous media are emerging in the literature. For instance, Mamajanov et al. observed that nucleic acid formed reversible secondary structures in anhydrous DESs, although the stability and shape of these structures differed from sequences stored in aqueous media.^17^ In another study, Mondal et al. demonstrated the preservation of DNA from salmon testes in glycerol-based DESs under harsh pH and thermal conditions.^18^ Furthermore, Gállego et al. demonstrated that a 32 base pair duplex maintained B-Form helical structure and exhibited higher thermal stability in DES compared to aqueous media.^19^ Their work is one of the first such examples of successful transfer of nucleic acid from an aqueous medium to a DES without compromising the structure and function of the nucleic acid polymer, thus bolstering the idea of DESs as a storage medium.

In addition to experimental work involving biomolecules and DESs, in recent times, there have been a number of computational modeling studies that focus on the interactions between DES and biomolecules to gain atomistic insights.^20^ Some of these modeling studies include reports on the solubility of a glycoside in DESs using COSMO-RS methodology^21^ and probing the mechanism for stabilization of nicotinamide adenine dinucleotide in DESs.^22^ Some studies have used molecular dynamics (MD) to explore the effect of water content in DESs on the activity of lipase;^23^ another study explored the molecular rigidity of DNA aptamers in DES.^24^ Although there has been some theoretical work on nucleic acid and DES interactions, most of it is focused on exploring the solvent structure,^25,26^ hydrogen-bond interactions,^27,28^ influence of water content on DES solvent structure,^29,30^ molecular diffusion in DESs,^31–33^ and DES–metal interactions.^34,35^ In this work, we have provided atomic insights into interactions between bases/base pairs in nucleic acids and trehaline (a mixture containing choline chloride and trehalose in a 1:1 molar ratio) DES using *ab initio* methods. The nontoxic nature of trehalose and its ability to solvate nucleic acids by functioning as an osmolyte make trehalose-based DES an ideal candidate for this study.^36,37^ Furthermore, aqueous solutions containing 10% trehalose are known to stabilize functional RNA at 4 °C for up to 10 months.^38^ Finally, encouraged by the work done by Xin et al. in which trehalose and choline chloride-based DES were used to preserve lysozyme, we were inspired to explore interactions between DES and nitrogenous bases of nucleic acids.^39^

We aim to investigate the thermochemistry, charge distribution, and structural motifs of trehaline with the free nitrogenous bases of nucleic acids (adenine, guanine, thymine, cytosine, and uracil) and their base pairs to provide comprehensive mechanistic insights into these interactions at an atomic level, as depicted in Figure 1. By delving into the unique properties of this natural DES system, we seek to gain a comprehensive mechanistic understanding of how each individual component of DES contributes to the stabilization of nucleic acids. Unraveling the intricate interplay between the DES constituents and the nucleic acid components will pave the way for the rational design of DES-based storage media capable of maintaining the structural and functional integrity of nucleic acids at ambient temperatures.

**Figure 1:**
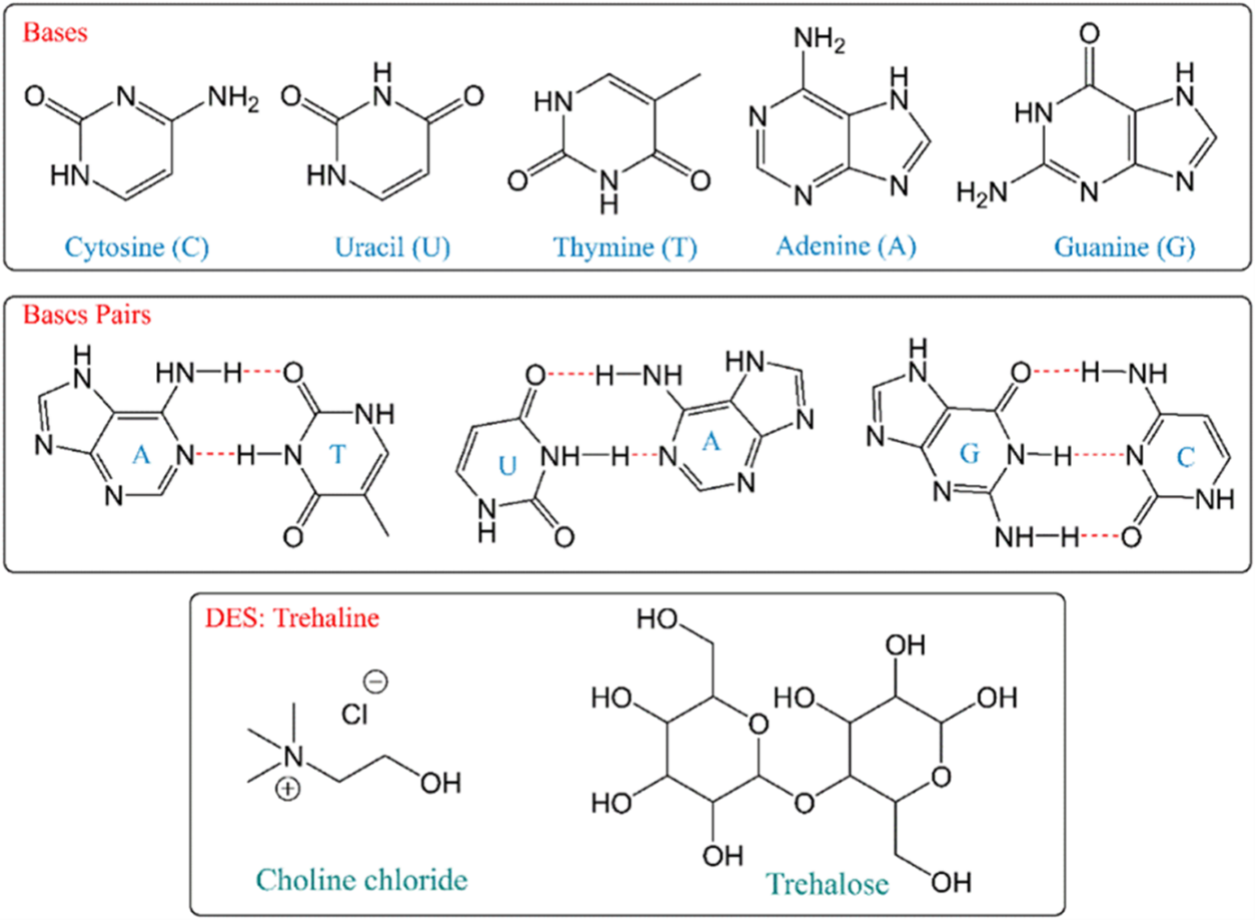
Chemical structures of model bases (top), base pairs of nucleic acids (middle), and DESs employed in this study (bottom).

## Experimental Methods

The experimental section including materials, preparation of DES, and characterization using NMR and FTIR has been thoroughly described in the Supporting Information.

### Computational Methods

Geometry optimization of the DES–base structures was carried out using the Gaussian 16W software package at the M06-2X/6-31++G­(d,p) theory and basis set.^40,41^ This method and basis set were successfully used in the prediction of geometry and thermochemistry in our previous work involving glucose- and trehalose-based DESs.^42^ The simulations were carried out using various starting orientations in which the DES was placed either in-plane or out-of-plane with nitrogenous bases. The starting orientations also included structures in which a base or a base pair was placed in proximity of the choline cation, or chloride anion, or disaccharide in the initial configurations (see Figure S1). All simulations were carried out with no symmetry restrictions in the singlet ground state. The absence of imaginary frequencies in the optimized structures indicated that the optimized structures were at minima. The dispersion corrections were accounted for using the method developed by Grimme within the framework of M06-2X functionals.^43^ Charge transfer was determined using CHarges from Electrostatic Potential maps using a Grid-based method (CHELPG).^44^ Bond order and Harmonic Oscillator Model of Aromaticity (HOMA index) were obtained using the Multiwfn software package.^45^

Changes in the thermochemical parameters such as energy of interaction (Δ*E*) and free energy (Δ*G*_F_) were determined for the DES–base and DES–base pair complexes using eq 1:

1 $$\Delta X_{\mathrm{System}}=X_{\mathrm{DES}+\mathrm{Base}}-X_{\mathrm{DES}}-X_{\mathrm{Base}}$$

where *X* represents interaction energy (*E*) and free energy (*G*_F_).

## Results and Discussion

The analysis of optimized geometries of solvent–solute systems allows us to investigate and potentially predict the nature of their atomic interactions such as hydrogen bonding, van der Waals forces, and electrostatic interactions. These interactions help to understand solvent–solute compatibility and subsequently the effect of these interactions on the structural stability of the solute. Our simulations have been targeted toward the influence of the solvent environment of the trehalose-based DES on base pairing of nitrogenous bases of nucleic acid: a critical feature in the structural stability of nucleic acids. During this process, we have compared interactions of DES with individual bases as well as base pairs to allow us to gauge the influence of DES on the pairing strength of these bases. The simulations were performed using the M06-2X/6-31++G­(d,p) method and basis set, which was successfully applied in our previous work on trehaline that helped to establish a relationship between the molecular structure and physical properties of the DES.^42^ We also successfully applied this method and basis set to investigate the structure–property relationship in ionic liquids containing heterocyclic aromatic moieties.^46^ Furthermore, Zhao and Truhlar have extensively described the efficiency of the M06-2X density functional in the prediction of thermochemistry and noncovalent interactions in ionic and heteroaromatic systems.^40^ Finally, the infrared spectrum of the optimized geometries of DES/base complexes computed at M06-2X/6-31++G­(d,p) was compared with the experimental spectrum. The scaling factor of 0.947 was used to correct the IR frequency calculations.^47^ A comparison between the experimental and computed IR spectra of the DES and base complexes indicates that the simulations were able to reproduce major features in the experimental IR spectrum (see Figure 2). Furthermore, the plots of peak positions in experimental and simulated IR spectra indicate a strong correlation (*R* ^2^ > 0.99), thus suggesting a reasonable agreement (see Figures S1 and S2).

**Figure 2:**
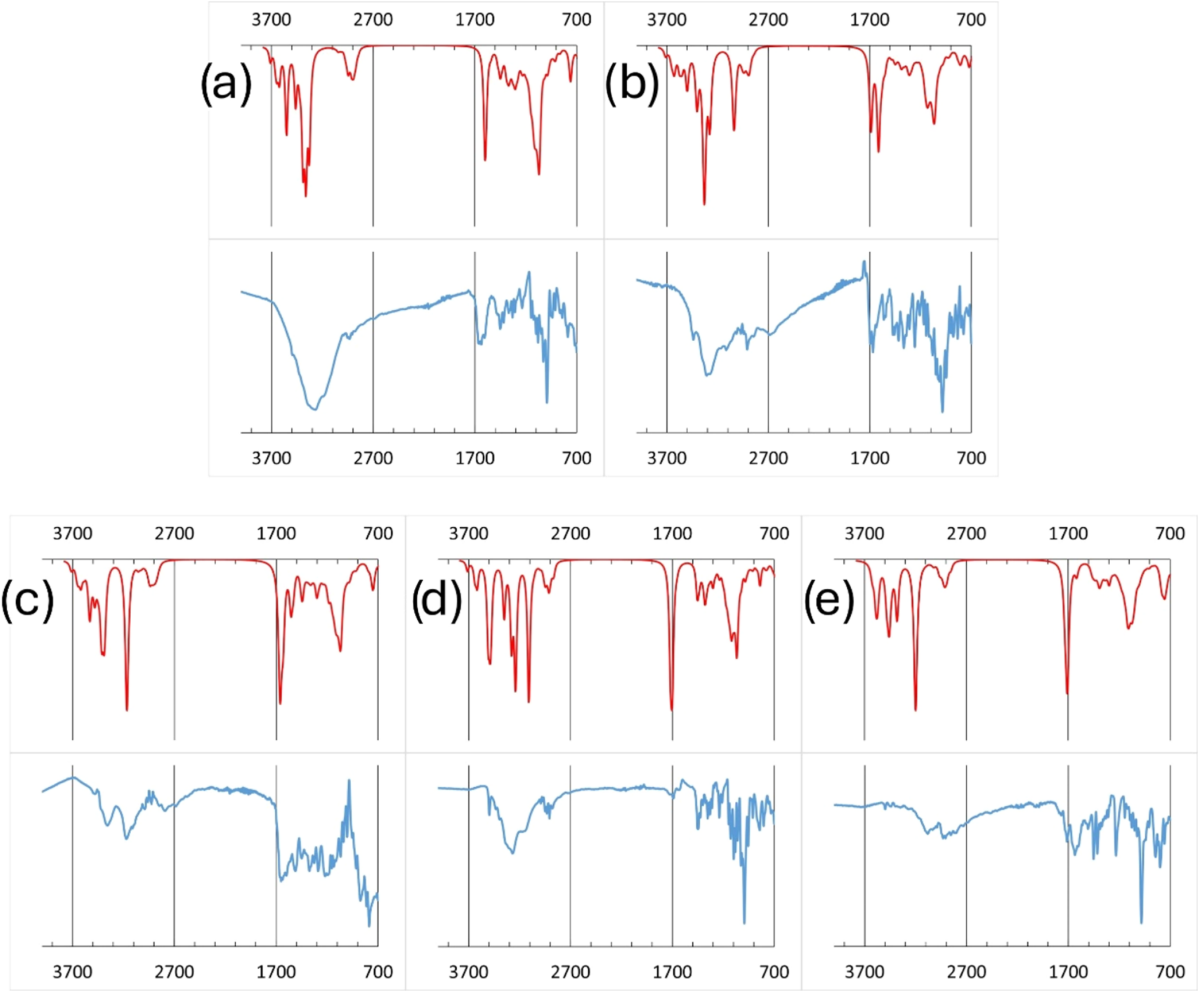
Comparison between experimental (blue) and computed (red) IR spectrum. (a) DES/adenine and (b) DES/guanine, (c) DES/cytosine, (d) DES/thymine, and (e) DES/uracil complexes. The IR spectrum computed at M06-2X/6-31++G­(d,p) theory was corrected using the scaling factor of 0.947 to match the experimental spectrum.

The simulation of DES with individual bases revealed that the size of the bases and their ability to hydrogen bond play a crucial role in their interactions with DES. It was observed that the purines (adenine and guanine), being larger in size due to a fused ring system with an extended π-system, interacted with DES components through C–H···π interactions as well as hydrogen bonding. However, the pyrimidines (cytosine, thymine, and uracil) interacted with DES primarily through hydrogen bonding. The pyrimidines act as a hydrogen bond donor through amide nitrogen (NH) to Cl^–^ with a bond length of 2.19 Å, whereas the carbonyl bond acts as a hydrogen bond acceptor through interaction with the hydroxyl group of trehalose with a hydrogen bond length of 1.90–1.94 Å. The NH moiety in pyrimidines also interacted with the hydroxyl oxygen of trehalose, resulting in a strong hydrogen bond (1.86 Å). Additionally, the pyrimidines interacted with acidic hydrogen atoms on the methyl groups in the choline cation through multiple weak CH···π interactions with distances ranging between 2.15 and 2.79 Å (Figure 3).

**Figure 3:**
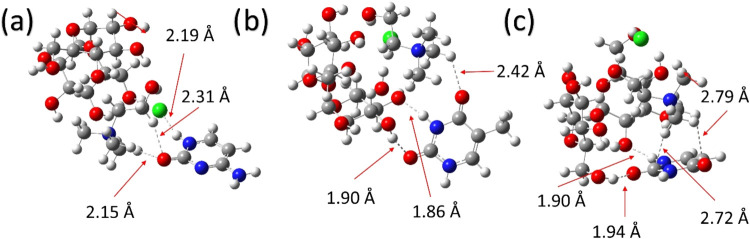
Optimized geometry of trehalose-based DES with pyrimidines: (a) cytosine, (b) thymine, and (c) uracil. Coloration of atoms: gray is carbon, white is hydrogen, red is oxygen, and blue is nitrogen.

In contrast, the DES interacted with purines primarily through C–H···π as well as hydrogen bonds, thus allowing higher degree interactions that resulted in cage-like structures. For instance, there is a single hydrogen-bonding interaction between adenine and Cl^–^ at 2.42 Å, whereas the methyl group of choline cation engages with the fused bicyclic ring of adenine through CH···π interactions at 2.43, 2.64, and 2.95 Å (Figure 4a). In guanine, the terminal amine acts as a hydrogen bond donor to the Cl^–^ at a distance of 2.15 Å, whereas the secondary amine engaged with the hydroxyl group on trehalose through a O–H···π interaction at 1.88 Å. Additionally, the hydrogen atom on the methyl group on choline interacted with carbonyl oxygen of guanine via a C–H···π interaction at a distance of 2.18 Å, whereas hydrogen of the methylene (−CH_2_−) group on the π-system of guanine interacted via the C–H···N interaction at a distance of 2.60 Å (Figure 4b).

**Figure 4:**
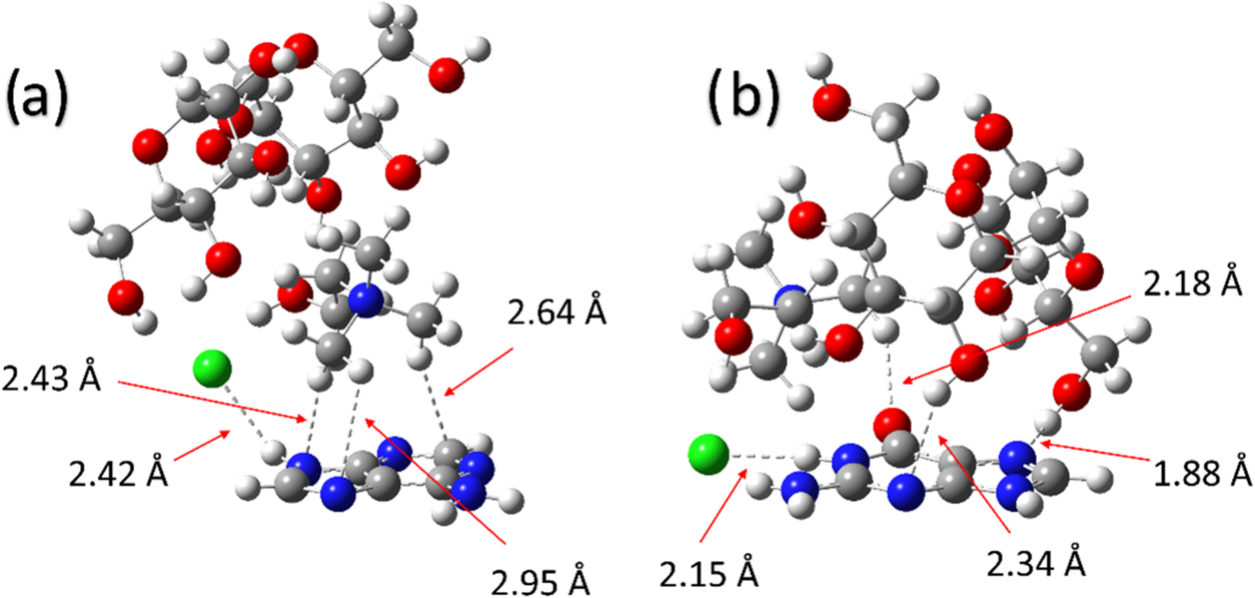
Optimized geometry of trehalose-based DES with purines: (a) adenine and (b) guanine. Coloration of atoms: gray is carbon, white is hydrogen, red is oxygen, and blue is nitrogen.

The simulations of free unpaired bases with DES served as a baseline for comparison with DES and base pair (BP) complexes such as adenine/thymine (AT), adenine/uracil (AU), and guanine/cytosine (GC). Among the three DES/base pair systems, the DES significantly altered the hydrogen-bonding architecture in AU, whereas the AT and GC systems largely retained their structure with minimal disruption by the DES (Figure 5). In the optimized geometry, it can be observed that the structural disruption of the base pair arrangement is a result of a combination of C–H···π interaction of the choline cation and hydrogen bonding from sugar and Cl^–^ with the AU base pairs. In the AT base pair, the bases in the base pair retain their planar configuration with respect to each other. This is primarily due to a strong CH···π and OH···π interactions between the methyl proton on the choline cation and the OH group of sugar, with the base at distacnes of 2.27 and 2.48 Å, respectively. This DES interaction led to a significant influence on the hydrogen bond between A and T, with the (N···HN) bond decreasing by 0.2–1.73 Å and the NH_2_···O bond increasing by 0.13–2.08 Å compared to free bases. Interestingly in AT base pair, the third interaction between the bases A and T, the CH···O=C shortened from 2.69 to 2.54 Å. In the AU base pair, the interaction with DES significantly disrupted the hydrogen bonds between bases; the hydrogen bond N···HN was disrupted by Cl^–^, and the length of the NH_2_···O hydrogen bond increased by 0.06 Å. In the AU base pair, the adenine interacts with trehalose through the CH···π interaction (2.45 Å) as well as through hydrogen bonds (2.03 Å), whereas uracil interacts with methyl protons on the choline at 2.33 Å. Interestingly, the GC base pair was minimally affected by DES and maintained its native base pair arrangement. Of the three hydrogen bonds between the base pairs, only one increased in length by 0.1 Å (NH_2_···O), while the N···HN bond was reduced in length by 0.01 Å, as can be observed by comparing Figures 5 and S4. This suggests that the GC base pair will resist structural changes in the presence of DES due to the presence of three hydrogen bonds in the base pair. The comparison between the DES interaction with the individual bases and base pairs revealed that purines like to interact primarily through the CH···π interaction, while the pyrimidines interact through hydrogen bonds. This is reflected in the base pair/DES interactions in which the CH···π dominated the purine component of the base pair side and hydrogen bonding on the pyrimidines. These interactions lead to partial disruption of AT base pair and complete disruption of AU base pair, yet the GC base pair with three hydrogen bonds was able to withstand the hydrogen bond disruption by DES.

**Figure 5:**
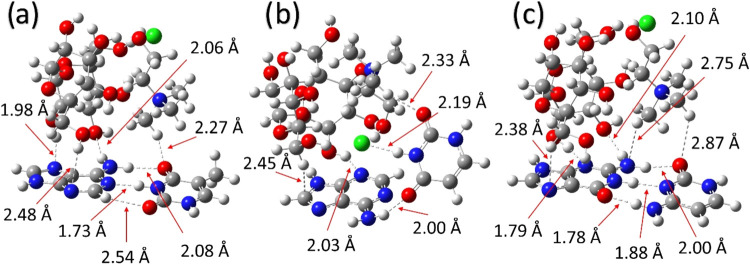
Optimized geometry of trehalose-based DES with base pairs: (a) adenine: thymine (AT), (b) adenine: uracil (AU), and (c) guanine: cytosine (GC). Coloration of atoms: gray is carbon, white is hydrogen, red is oxygen, and blue is nitrogen.

### Aromaticity and Bond Order Analysis

In nucleic acids, π···π stacking between adjacent aromatic base pairs is one of the important factors that render stability to the double helical structure of the nucleic acids. Therefore, it is important to understand the influence of DES on the aromatic character of the bases. In this work, the influence of DES interaction on the aromatic character of the bases was analyzed by the Harmonic Oscillating Model of Aromaticity (HOMA) index using the Multiwfn software package.^45^ The HOMA index is an estimate of the aromatic character, which is derived from the geometrical parameters of a molecule. A HOMA index value of 1 indicates a perfectly aromatic nature of a molecule (i.e., benzene), whereas a molecule with a value of 0 is not aromatic.

The interaction with DES led to a marked increase in HOMA index values of the bases. Adenine and cytosine experienced 0.01 and 0.1 units gain in the HOMA value, respectively, whereas the pyrimidines, guanine, thymine, and uracil, gained 0.086, 0.053, and 0.068 units in HOMA values, respectively (Table S1). The AT and GC base pairs, which partially or completely retained the base pairing, gained aromatic character by 0.050 and 0.032 units, respectively, whereas the AU base pair, which was disrupted by the DES, experienced a loss in aromatic character by 0.037 units (Table 1). In the case of AT and GC base pairs, the gain in the aromatic character of the heterocyclic rings primarily arises due to the dominance of C–H···π interaction with the sugar and choline (Figure 4). In the case of the AU base pair, the reduction in aromatic character is due to the strong interaction of Cl^–^ with the uracil in the base pair, as indicated by the increase in the electron density on Cl^–^, see Table 3 on charge transfer.

**Table 1:** Calculated HOMA Index Values Indicate the Change in the Aromatic Character of the Base Pairs AT, GC, and AU in the Presence and Absence of DES

	HOMA values of bases in the BP		
base pair (BP)	no DES (X)	DES (Y)	ΔHOMA (X – Y)
AT	1.4590	1.5088	+0.0498
GC	1.5280	1.5598	+0.0318
AU	1.4922	1.4556	–0.0366

Further, we performed multicenter bond order (MBO) analysis to gauge the effects of DES on the hydrogen bonding between the bases in the base pairs, as it is an effective means to quantify the strength of a noncovalent interaction between molecules. Table 2 quantifies the strength of hydrogen bonds between bases in the base pairs in the presence and absence of DES. The MBO analysis indicates that the hydrogen bonding between base pair AT was weakened and that between AU was significantly disrupted in the presence of DES, as indicated by the lowering of the bond order values. This result is consistent with the geometry analysis. Interestingly, the presence of DES resulted in the strengthening of the hydrogen bonding between bases in the GC base pair. The strengthening of the G and C interactions correlates with an increase in the electron density and aromatic character of the GC base pair in the presence of DES. Additionally, the presence of three hydrogen bonds in GC as opposed to two in AT and AU base pairs makes the GC base pair significantly more resistant to the disruption of base pairing caused by hydrogen bond disruption by the DES.

**Table 2:** Multicenter Bond Order Analysis of Hydrogen Bonds between Bases in the Base Pairs in the Presence and Absence of DESs

base pairs	hydrogen bonds between base pairs	base pairs (no DES)	base pairs (with DES)
**A**T	**NH**···O	0.0413	0.0066
	**N**···HN	0.1616	0.0801
**A**U	**NH**···O	0.0572	0.0047
	**N**···HN	0.1565	interaction disrupted
**G**C	**O**···HN	0.0707	0.1004
	**NH**···N	0.1050	0.1551
	**NH**···O	0.0605	0.0822

### Charge Transfer

Interactions between molecular species result in the redistribution of electron density between interacting partners. The extent of electron density redistribution is an indicator of the strength of interactions between molecular species. In our previous study, we investigated charge transfer between various components of the trehalose-based DES using the CHELPG method. In that work, we found that the choline cation donates significant electron density to trehalose through the hydrogen atoms on the choline headgroup.^42^ Similarly, in this work, we performed an investigation of charge transfer between DES and bases/base pairs complexes. This analysis will provide a correlation between the direction of charge transfer and is associated with the type of interactions between DES and bases/base pairs. The analysis revealed a stark difference in charge transfer purines versus pyrimidine in DES. The purines lost electron density to DES as indicated by a positive charge, while the pyrimidines gained electron density from DES as suggested by a negative charge (Table S2). This disparity in the direction of the charge transfer is due to the difference in the way DES interacts with purines versus pyrimidines. In DES/purines complexes, the DES interacts with the fused ring structure with an electron-rich extended π-surface through choline cation (C–H···π) and trehalose (O···π) interactions that result in the loss of electron density. However, in DES/pyrimidines complexes, the DES and pyrimidines primarily interacted through hydrogen bonding via hydroxyl groups on trehalose with the carbonyl oxygen (C=O) and amide nitrogen (NH) on pyrimidines, resulting in the net gain of electron density to the DES components (Table S2).

In base pairs, AT and GC lost electron density to DES by 0.097e and 0.086e, respectively, whereas the AU base pair gained electron density by 0.024e (Table 3). In AT and GC base pairs, the loss in the electron density is a result of interaction of DES with the electron-rich π-system of the base pairs via C–H···π and OH···π interactions between choline and trehalose components of DES. In the case of the AU base pair, the DES is involved in hydrogen bonding with the electronegative atoms on edges of the bases and results in electron density transfer from DES as evident from Figure 4. Interestingly, it can be noted in Table 3 that the choline cation in the DES/AU complex significantly lost electron density, which is redistributed to other fragments of the complex with AU base pair gaining negative charge by 0.024e. Overall, the interaction of DES with the π-system of base pairs resulted in the loss of electron density in base pairs, whereas the interaction of DES with the edge of base pair resulted in an increase in electron density and disruption of the base pairing.

**Table 3:** Electronic Charges Calculated for the DES/Base Pair Complexes Using the CHELPG Method

individual components	free DES	AT/DES	GC/DES	AU/DES
trehalose	–0.068	–0.119	–0.154	–0.108
choline	0.829	0.790	0.810	0.926
chloride	–0.761	–0.768	–0.742	–0.794
base pair		0.097	0.086	–0.024
total charge	0.00	0.00	0.00	0.00

### Thermochemistry

The quantification of change in thermochemical parameters such as free energy and interaction energy helps us to understand the strength of the interactions between different molecular species. The change in the free energy (Δ*G*_F_) and total energy (Δ*E*) associated with the interaction of DES with the model bases and base pairs were determined using eq 1. The −Δ*G*_F_ values indicate favorable interactions of DES with bases and base pairs. Interestingly, the comparison of Δ*G*_F_ and Δ*E* values indicates that the DES/base complexes have significantly favorable Δ*G*_F_ values over base pair complexes (Tables 4 and 5). This is due to the ability of the free bases to form a higher number of interactions with the DES compared to the base pairs. On the other hand, the Δ*E* values for all of the DES/base and DS/base pair systems remained within the range of −19.04 to −24.04 kcal/mol, with the exception of the guanine/DES system, which had a value of −32.08 kcal/mol. This is due to the interactions of guanine with all three components of DES, which are absent in other DES/base systems. In the DES/base pair systems, the GC/DES system displayed Δ*G*_F_ and Δ*E* values of −7.49 and −24.19, respectively, which is more favorable than the DES/AT and DES/AU systems by approximately 2.0 kcal/mol. This discrepancy is due to the retention of the planar arrangement in the GC base pair, along with an increase in the bond order for the three hydrogen bonds between bases in the presence of DES (Table 2). The planar arrangement is distorted by DES in the case of AT and AU base pairs, along with the weakening of hydrogen bonds by the DES interaction, resulting in relatively less favorable Δ*G* and Δ*E* values compared to the GC base pair. Although favorable, these thermochemical values are associated with strengthening of the GC base pair and weakening in the case of AT and AU base pairs. This led us to query the ability of the unpaired bases to pair in the presence of DES in the media. To address this question, we estimated the difference in the free energy (ΔΔ*G*_F_) between the DES/base and DES/base/pair complexes using eq 1 in the Computational Methods section. It was found that the ΔΔ*G*_F_ was unfavorable for the formation of base pairs AU and GC in DES by +2.77 and +6.44 kcal/mol, respectively, thus indicating the high cost of base pairing in DES once they are unpaired (Figure 6). However, for the AT base pair, the value was marginally lower by −0.76 kcal/mol, indicating a favorable pairing between A and T in DES. The more favorable thermodynamics of free bases compared to base pairs may be attributed to the greater accessibility of deep eutectic solvents (DES) to nitrogen atoms, specifically N9 in purines and N1 in pyrimidines which would otherwise be engaged with the sugar and phosphate backbone in nucleic acids. Overall, the thermodynamic studies favor AT and GC base pairs. It is a thermodynamically uphill task for unpaired bases to engage in complementary hydrogen bonding in choline chloride and trehalose-based DES. Although this study has examined the molecular interactions and thermodynamic properties of short-range structural features, further computational analysis and extrapolation are required to evaluate the potential of DES for the storage of mRNA vaccines containing sequences of approximately 4250 nucleotides. Moreover, there remains a significant opportunity to investigate long-range interactions by employing molecular dynamics (MD) simulations to predict DNA/RNA folding, base pair dynamics, and stacking behavior within DES environments.

**Table 4:** Changes in the Free Energy and Interaction Energy for Choline Chloride/Trehalose-Based DES and the Model Bases Found in Nucleic Acids

	DES/A	DES/G	DES/C	DES/T	DES/U
Δ*E* (kcal/mol)	–20.79	–32.08	–24.04	–19.04	–21.82
Δ*G*_F_ (kcal/mol)	–4.34	–15.96	–11.91	–5.02	–10.04

**Table 5:** Changes in the Free Energy and Interaction Energy for Choline Chloride/trehalose-based DES and the Model Base Pairs Found in Nucleic Acids

	DES/AT	DES/AU	DES/GC
Δ*E* (kcal/mol)	–19.36	–20.27	–24.19
Δ*G* (kcal/mol)	–3.25	–4.42	–7.49

**Figure 6:**
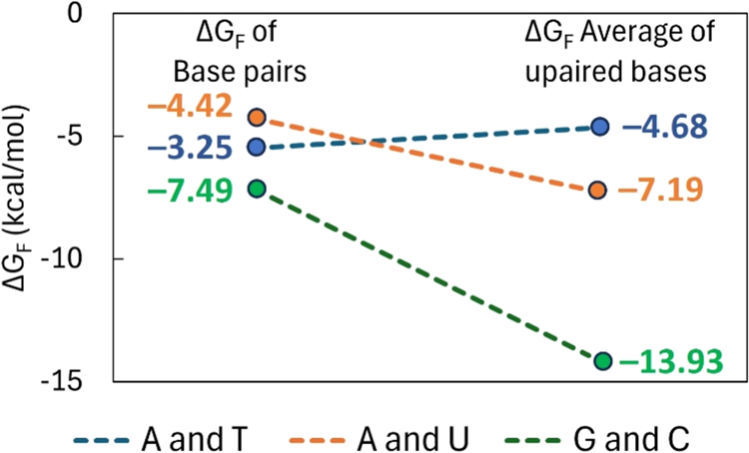
Average change in the free energy for DES/base complexes involved in base pairing.

## Conclusion

There is a critical need to eliminate or improve this vaccine coverage gap by minimizing the costly and labor-intensive cold chain process, thereby providing equitable access to these life-saving therapeutics. In the case of unpaired bases, purines interacted with DES through C–H···π interactions as well as hydrogen bonding, facilitating stronger interactions leading to cage-like structures. On the other hand, pyrimidines primarily interact through hydrogen bonding alone. In DES/base pair complexes, the AT base pair exhibited significant weakening and loss of planarity due to C–H···π interactions between choline, sugar, and the bases. Conversely, the AU base pair was completely disrupted, attributed to additional hydrogen bonding with chloride ions. Interestingly, the GC base pair retained its base pairing integrity, with hydrogen bonds between bases strengthened in the presence of DES, as evidenced by bond distance and bond order data. However, the GC base pair showed reduced aromatic character due to the dominance of C–H···π interactions in the DES/GC complex. Analysis using the HOMA index revealed increased aromaticity in unpaired bases and base pairs AT and AU owing to a high number of hydrogen-bonding interactions. Conversely, the DES/GC complex showed a reduction in aromatic character. Further analysis using molecular bond order (MBO) indicated a reduction in bond order for hydrogen bonds between AT and AU, while the GC base pair exhibited strengthened bonds in the presence of DES. Charge transfer analysis revealed that purines (A and G) engaging with DES through C–H···π interactions lost electron density, while pyrimidines, which primarily hydrogen bonded with DES, gained electron density. Interestingly, these trends were reversed in DES/base pair complexes. Thermochemical analysis showed that the Δ*G*_F_ and Δ*E* values for interactions of DES with unpaired bases and base pairs were favorable. Further thermochemical analysis revealed that the formation of the base pairs from the unpaired bases is a thermodynamically unfavorable process. While this study has delved into the molecular interactions and thermodynamics of choline chloride/trehalose-based DES with model bases in nucleic acids, focusing on short-range structures, there remains an opportunity to investigate interactions of DES with the sugar and phosphate backbone. Such exploration could provide further insights into the interactions of DES with nucleic acids and their implications on structure and stability in nucleic acids in these novel solvent environments.

## Supplementary Material



## Data Availability

The data that supports the findings of this study are available within the article [and its Supporting Information].

## Notes

Experimental data and Cartesian coordinates. (PDF)

## References

[1] Moore S., Hill E. M., Dyson L., Tildesley M. J., Keeling M. J. (2022). Retrospectively Modeling the Effects of Increased Global
Vaccine Sharing on the COVID-19 Pandemic. Nat.
Med..

[2] He W., Zhan T., Han H., Xu Y. (2024). Optimization of Deep
Eutectic Solvents Enables Green and Efficient Cryopreservation. Langmuir.

[3] Abbott A. P., Boothby D., Capper G., Davies D. L., Rasheed R. K. (2004). Deep Eutectic
Solvents Formed between Choline Chloride and Carboxylic Acids: Versatile
Alternatives to Ionic Liquids. J. Am. Chem.
Soc..

[4] Paiva A., Craveiro R., Aroso I., Martins M., Reis R. L., Duarte A. (2014). Natural Deep Eutectic Solvents – Solvents for
the 21st Century. ACS Sustainable Chem. Eng..

[5] Kist J. A., Zhao H., Mitchell-Koch K. R., Baker G. A. (2021). The Study and Application
of Biomolecules in Deep Eutectic Solvents. J.
Mater. Chem. B.

[6] Mbous Y. P., Hayyan M., Wong W. F., Looi C. Y., Hashim M. A. (2017). Unraveling
the Cytotoxicity and Metabolic Pathways of Binary Natural Deep Eutectic
Solvent Systems. Sci. Rep..

[7] Yadav A., Pandey S. (2014). Densities and Viscosities
of (Choline Chloride + Urea)
Deep Eutectic Solvent and Its Aqueous Mixtures in the Temperature
Range 293.15 to 363.15 K. J. Chem. Eng. Data.

[8] O’Neill M. K., Piligian B. F., Olson C. D., Woodruff P. J., Swarts B. M. (2017). Tailoring
Trehalose for Biomedical and Biotechnological Applications. Pure Appl. Chem..

[9] Ahlgren K., Olsson C., Ermilova I., Swenson J. (2023). New Insights into the
Protein Stabilizing Effects of Trehalose by Comparing with Sucrose. Phys. Chem. Chem. Phys..

[10] Vinciguerra D., Gelb M. B., Maynard H. D. (2022). Synthesis
and Application of Trehalose
Materials. JACS Au.

[11] Wang X., Liu M., Peng F., Ding X. (2021). Hydrophobic Magnetic Deep Eutectic
Solvent: Synthesis, Properties, and Application in DNA Separation. J. Chromatogr. A.

[12] Zhang H., Wang Y., Zhou Y., Xu K., Li N., Wen Q., Yang Q. (2017). Aqueous Biphasic Systems Containing
PEG-Based Deep
Eutectic Solvents for High-Performance Partitioning of RNA. Talanta.

[13] Belviso B. D., Perna F. M., Carrozzini B., Trotta M., Capriati V., Caliandro R. (2021). Introducing
Protein Crystallization in Hydrated Deep
Eutectic Solvents. ACS Sustainable Chem. Eng..

[14] Lehmann C., Sibilla F., Maugeri Z., Streit W. R., María P. D. de., Martinez R., Schwaneberg U. (2012). Reengineering CelA2 Cellulase for
Hydrolysis in Aqueous Solutions of Deep Eutectic Solvents and Concentrated
Seawater. Green Chem..

[15] Silva N. H. C. S., Pinto R. J. B., Freire C. S. R., Marrucho I. M. (2016). Production of Lysozyme
Nanofibers Using Deep Eutectic Solvent Aqueous Solutions. Colloids Surf., B.

[16] Liu Y., Wang Y., Dai Q., Zhou Y. (2016). Magnetic Deep Eutectic
Solvents Molecularly Imprinted Polymers for the Selective Recognition
and Separation of Protein. Anal. Chim. Acta.

[17] Mamajanov I., Engelhart A. E., Bean H. D., Hud N. V. (2010). DNA and RNA in Anhydrous
Media: Duplex, Triplex, and G-Quadruplex Secondary Structures in a
Deep Eutectic Solvent. Angew. Chem., Int. Ed..

[18] Mondal D., Sharma M., Mukesh C., Gupta V., Prasad K. (2013). Improved Solubility
of DNA in Recyclable and Reusable Bio-Based Deep Eutectic Solvents
with Long-Term Structural and Chemical Stability. Chem. Commun..

[19] Gállego I., Grover M. A., Hud N. V. (2015). Folding
and Imaging of DNA Nanostructures
in Anhydrous and Hydrated Deep-Eutectic Solvents. Angew. Chem., Int. Ed..

[20] Wagle D. V., Adhikari L., Baker G. A. (2017). Computational
Perspectives on Structure,
Dynamics, Gas Sorption, and Bio-Interactions in Deep Eutectic Solvents. Fluid Phase Equilib..

[21] Jeliński T., Cysewski P. (2018). Application of a Computational Model
of Natural Deep
Eutectic Solvents Utilizing the COSMO-RS Approach for Screening of
Solvents with High Solubility of Rutin. J. Mol.
Model..

[22] Radović M., Hok L., Panić M., Bubalo M. C., Vianello R., Vinković M., Redovniković I. R. (2022). Deep Eutectic Solvents as a Stabilising
Medium for NAD Coenzyme: Unravelling the Mechanism behind Coenzyme
Stabilisation Effect. Green Chem..

[23] Shehata M., Unlu A., Sezerman U., Timucin E. (2020). Lipase and Water in
a Deep Eutectic Solvent: Molecular Dynamics and Experimental Studies
of the Effects of Water-In-Deep Eutectic Solvents on Lipase Stability. J. Phys. Chem. B.

[24] Pal S., Paul S. (2019). Effect of Hydrated
and Nonhydrated Choline Chloride–Urea Deep
Eutectic Solvent (Reline) on Thrombin-Binding G-Quadruplex Aptamer
(TBA): A Classical Molecular Dynamics Simulation Study. J. Phys. Chem. C.

[25] Rain M. I., Iqbal H., Saha M., Ali M. A., Chohan H. K., Rahman M. S., Halim M. A. (2021). A Comprehensive
Computational and
Principal Component Analysis on Various Choline Chloride-Based Deep
Eutectic Solvents to Reveal Their Structural and Spectroscopic Properties. J. Chem. Phys..

[26] Das A., Das S., Biswas R. (2015). Density Relaxation
and Particle Motion Characteristics
in a Non-Ionic Deep Eutectic Solvent (Acetamide + Urea): Time-Resolved
Fluorescence Measurements and All-Atom Molecular Dynamics Simulations. J. Chem. Phys..

[27] Perkins S. L., Painter P., Colina C. M. (2014). Experimental
and Computational Studies
of Choline Chloride-Based Deep Eutectic Solvents. J. Chem. Eng. Data.

[28] Wagle D. V., Deakyne C. A., Baker G. A. (2016). Quantum
Chemical Insight into the
Interactions and Thermodynamics Present in Choline Chloride Based
Deep Eutectic Solvents. J. Phys. Chem. B.

[29] Kalhor P., Zheng Y.-Z., Ashraf H., Cao B., Yu Z.-W. (2020). Influence
of Hydration on the Structure and Interactions of Ethaline Deep-Eutectic
Solvent: A Spectroscopic and Computational Study. ChemPhysChem.

[30] Pandey A., Pandey S. (2014). Solvatochromic Probe Behavior within Choline Chloride-Based
Deep Eutectic Solvents: Effect of Temperature and Water. J. Phys. Chem. B.

[31] Pour S. B., Sardroodi J. J., Ebrahimzadeh A. R. (2021). The Study of Structure and Interactions
of Glucose-Based Natural Deep Eutectic Solvents by Molecular Dynamics
Simulation. J. Mol. Liq..

[32] Faraone A., Wagle D. V., Baker G. A., Novak E. C., Ohl M., Reuter D., Lunkenheimer P., Loidl A., Mamontov E. (2018). Glycerol Hydrogen-Bonding
Network Dominates Structure and Collective Dynamics in a Deep Eutectic
Solvent. J. Phys. Chem. B.

[33] Wagle D. V., Baker G. A., Mamontov E. (2015). Differential
Microscopic Mobility
of Components within a Deep Eutectic Solvent. J. Phys. Chem. Lett..

[34] Zhang C., Jia Y., Jing Y., Wang H., Hong K. (2014). Main Chemical Species
and Molecular Structure of Deep Eutectic Solvent Studied by Experiments
with DFT Calculation: A Case of Choline Chloride and Magnesium Chloride
Hexahydrate. J. Mol. Model..

[35] Atilhan M., Aparicio S. (2016). Deep Eutectic Solvents
on the Surface of Face Centered
Cubic Metals. J. Phys. Chem. C.

[36] Spiess A.-N., Mueller N., Ivell R. (2004). Trehalose Is a Potent PCR Enhancer:
Lowering of DNA Melting Temperature and Thermal Stabilization of Taq
Polymerase by the Disaccharide Trehalose. Clin.
Chem..

[37] Bezrukavnikov S., Mashaghi A., van Wijk R. J., Gu C., Yang L. J., Gao Y. Q., Tans S. J. (2014). Trehalose Facilitates
DNA Melting:
A Single-Molecule Optical Tweezers Study. Soft
Matter.

[38] Jones K. L., Drane D., Gowans E. J. (2007). Long-Term Storage of DNA-Free RNA
for Use in Vaccine Studies. BioTechniques.

[39] Xin R., Qi S., Zeng C., Khan F. I., Yang B., Wang Y. (2017). A Functional
Natural Deep Eutectic Solvent Based on Trehalose: Structural and Physicochemical
Properties. Food Chem..

[40] Zhao Y., Truhlar D. G. (2008). The M06 Suite of
Density Functionals for Main Group
Thermochemistry, Thermochemical Kinetics, Noncovalent Interactions,
Excited States, and Transition Elements: Two New Functionals and Systematic
Testing of Four M06-Class Functionals and 12 Other Functionals. Theor. Chem. Acc..

[41] Frisch, M. J. ; Trucks, G. W. ; Schlegel, H. B. ; Scuseria, G. E. ; Robb, M. A. ; Cheeseman, J. R. ; Scalmani, G. ; Barone, V. ; Petersson, G. A. ; Nakatsuji, H. ; Li, X. ; Caricato, M. ; Marenich, A. V. ; Bloino, J. ; Janesko, B. G. ; Gomperts, R. ; Mennucci, B. ; Hratchian, H. P. ; Ortiz, J. V. ; Izmaylov, A. F. ; Sonnenberg, J. L. ; Williams-Young, D. ; Ding, F. ; Lipparini, F. ; Egidi, F. ; Goings, J. ; Peng, B. ; Petrone, A. ; Henderson, T. ; Ranasinghe, D. ; Zakrzewski, V. G. ; Gao, J. ; Rega, N. ; Zheng, G. ; Liang, W. ; Hada, M. ; Ehara, M. ; Toyota, K. ; Fukuda, R. ; Hasegawa, J. ; Ishida, M. ; Nakajima, T. ; Honda, Y. ; Kitao, O. ; Nakai, H. ; Vreven, T. ; Throssell, K. ; Montgomery Jr, J. A. ; Peralta, J. E. ; Ogliaro, F. ; Bearpark, M. J. ; Heyd, J. J. ; Brothers, E. N. ; Kudin, K. N. ; Staroverov, V. N. ; Keith, T. A. ; Kobayashi, R. ; Normand, J. ; Raghavachari, K. ; Rendell, A. P. ; Burant, J. C. ; Iyengar, S. S. ; Tomasi, J. ; Cossi, M. ; Millam, J. M. ; Klene, M. ; Adamo, C. ; Cammi, R. ; Ochterski, J. W. ; Martin, R. L. ; Morokuma, K. ; Farkas, O. ; Foresman, J. B. ; Fox, D. J. Gaussian 16, Revision 01B; Gaussian, Inc.: Wallingford, CT, 2016.

[42] Recker E.
A., Hardy D., Anderson G. I., Mirjafari A., Wagle D. V. (2021). Covalently Linked
Hydrogen Bond Donors: The Other Side
of Molecular Frustration in Deep Eutectic Solvents. J. Chem. Phys..

[43] Grimme S. (2006). Semiempirical
GGA-Type Density Functional Constructed with a Long-Range Dispersion
Correction. J. Comput. Chem..

[44] Breneman C. M., Wiberg K. B. (1990). Determining Atom-Centered
Monopoles from Molecular
Electrostatic Potentials. The Need for High Sampling Density in Formamide
Conformational Analysis. J. Comput. Chem..

[45] Lu T., Chen F. (2012). Multiwfn:
A Multifunctional Wavefunction Analyzer. J.
Comput. Chem..

[46] Anderson G. I., Hardy D., Hillesheim P. C., Wagle D. V., Zeller M., Baker G. A., Mirjafari A. (2023). Anticancer Agents as Design Archetypes:
Insights into the Structure–Property Relationships of Ionic
Liquids with a Triarylmethyl Moiety. ACS Phys.
Chem. Au.

[47] Rodrigues-Oliveira A. F., Ribeiro F. W. M., Cervi G., Correra T. C. (2018). Evaluation of Common
Theoretical Methods for Predicting Infrared Multiphotonic Dissociation
Vibrational Spectra of Intramolecular Hydrogen-Bonded Ions. ACS Omega.

