# Supplementary material for: Mechanistic Insights into the Stabilizing Role of Deep Eutectic Solvents for Nucleic Acids: An In Silico Analysis
Source: J Phys Chem B. 2025 Jun 3;129(23):5674–82. doi: 10.1021/acs.jpcb.5c00799 (PMC12169689; doi:10.1021/acs.jpcb.5c00799)
Supplement: Supplementary file 1 [file jp5c00799_si_001.pdf]

Supporting Information

Computational Inquiry into Deep Eutectic Solvents as a Medium for Long-Term Nucleic Acid Preservation

David E. Hardy†, Adam Albert†, Zachary J. Metott‡, Arsalan Mirjafari‡*, Durgesh V. Wagle†*

†Department of Chemistry and Physics, Florida Gulf Coast University, Fort Myers, Florida 33965, United States

‡Department of Chemistry, State University of New York at Oswego, Oswego, New York 13126, United States

Email: *arsalan.mirjafari@oswego.edu and dwagle@fgcu.edu*

Table of Contents

Experimental Methods……………………….………………………………………………..S2

Starting structure for DES/Bases complexes and optimized geometry of base pairs…………....S3

Tables on HOMA and Charge transfer analysis ………………………………………………...S4

Cartesian coordinates of DES, bases, DES/bases and DES/base pair complexes…………S4–S21

Materials: Choline Chloride (99%, Cat. No: 110290010), Adenine (99%, Cat: A14906.14), Uracil (99+%, Cat: 157300250), Cytosine (99+%, Cat: 161760050), Thymine (99%, Cat: 157850050) wer bought from Thermo Fisher scientific. Guanine (>98%, CAS RN: 73-40-5) and D- (+) Trehalose Dihydrate (98%, CAS RN: 6138-23-4) were bought from TIC chemicals. Deionized water (18.2 MW×cm, Millipore system) was used for all experimental procedures.

Preparation of DES Mixtures:

The Deep Eutectic Solvent (DES) was prepared using choline chloride (5g, 0.036 moles) and trehalose (12.32g) in a 1:1 molar ratio. The mixture was transferred into a round-bottom flask and heated to 95˚C while subsequentially stirred under a vacuum at 90 rpm for three hours to reach optimal homogeneity, using a rotary evaporator (Buchi Rotavapor R-300).

Choline Chloride and Trehalose (1:1) mixture: ^{1}H NMR (400 MHz, DMSO-D_6) δ 2.85 (d, 2H), 1.72 (m, 2H), 1.53–1.38 (m, 8H), 1.32–1.25 (dd, 2H), 1.19–1.7 (dd, 2H), 1.10–0.90 (dt, 2H), 0.86 (s, 9H).

DES and nucleobase mixture: The DES and nucleobase mixtures were prepared by mixing the DES and the bases such that choline chloride, trehalose and nucleobases were in in 1:1:1 mole ratio in a round bottom flask.

Characterization Techniques:

Fourier-transform infrared spectroscopy (FTIR) was carried out using a Jasco FT/IR-4700 spectrometer microscope to analyze the molecular interactions between the components of each DES. Spectra were recorded ranging from 4000-500 cm^{-1} with a resolution of 4 cm^{-1}. There were 64 scans collected for each sample, with noise elimination, H_2O reduction, and CO_2 reduction applied. Baseline corrections and smoothing were performed to enhance the spectra accuracy.

Nuclear magnetic resonance (NMR) spectroscopy was performed using a Joel JNM-ECZ400S 400 MHz NMR spectrometer.

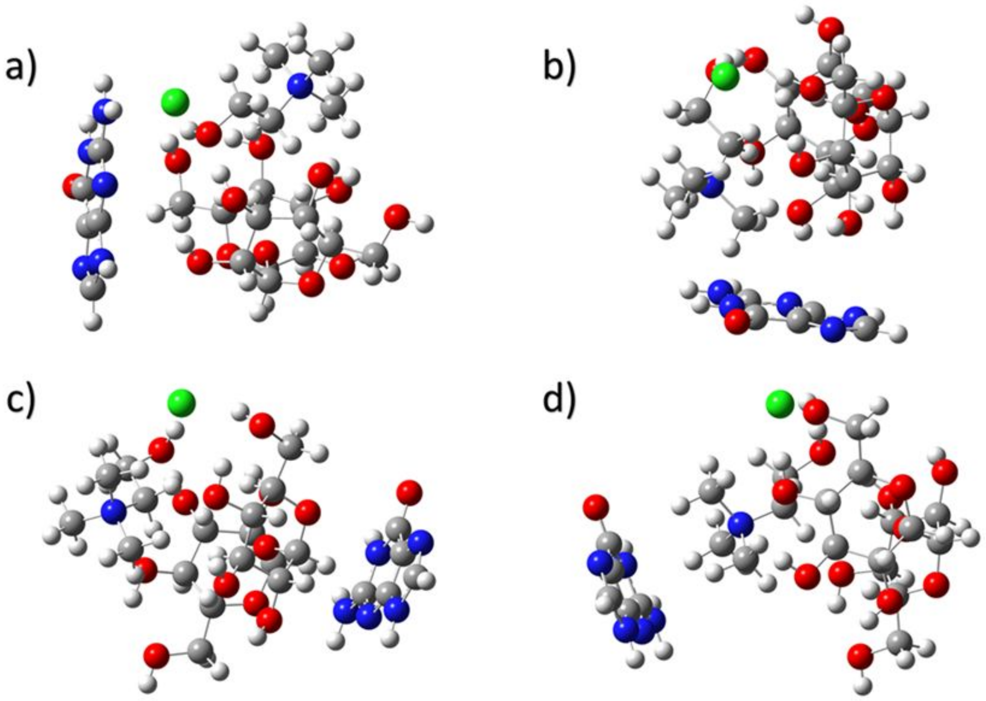

Figure S1. Starting geometries of bas/ DES cluster displaying positioning of base around the trehalose/choline chloride-based DES.

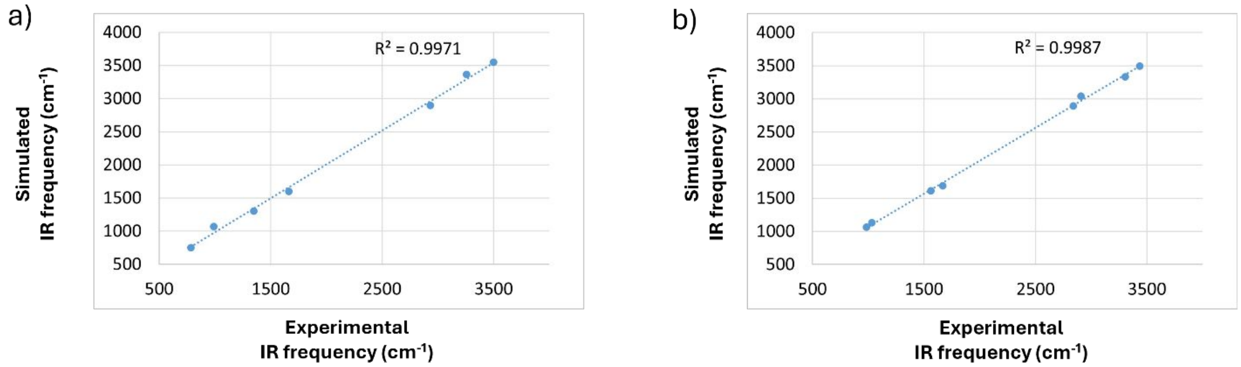

Figure S2. Correlation plots of experimental IR and simulated IR for a) adenine/DES and b) guanine/DES complexes.

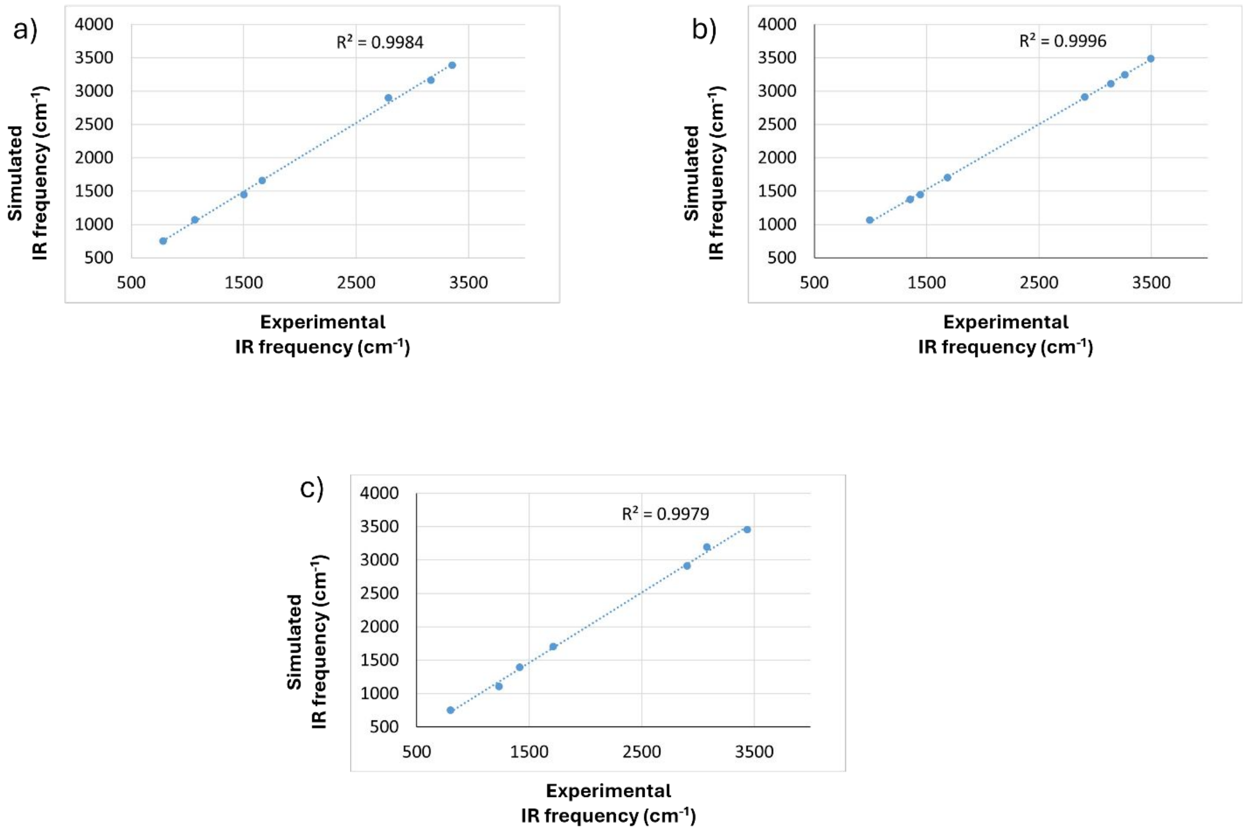

Figure S3. Correlation plots of experimental IR and simulated IR for a) cytosine/DES and b) Thiamine/DES, and c) uracil/DES complexes.

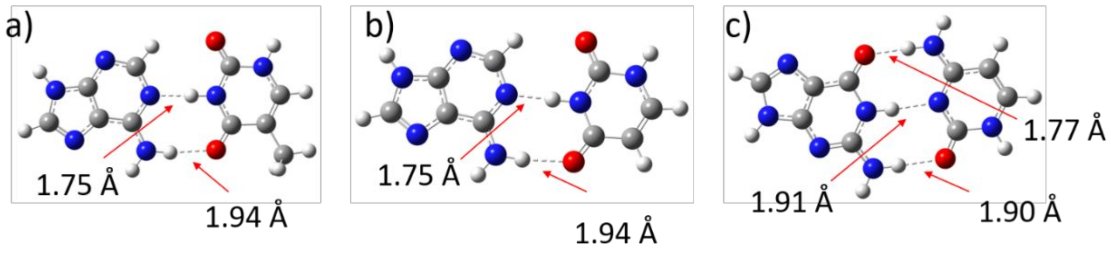

Figure S4. Optimized geometry of base pairs a) adenine/thymine, b) adenine uracil and c) Guanine/cytosine at M062x/6-31++G(d,p).

Bases	HOMA $_{(no\ DES)}$ (X)	HOMA $_{(DES)}$ (Y)	Δ HOMA (X–Y)
**A**	0.9200	0.9315	+0.0115
**G**	0.7392	0.8252	+0.0860
**C**	0.6799	0.7859	+0.1060
**T**	0.4751	0.5279	+0.0528
**U**	0.5109	0.5778	+0.0669

Table S1. The calculated HOMA index values indicate the change in the aromatic character of the bases Adenine (A), Guanina (G), Cytosine (C), thymine (T), and uracil (U) in presence and absence of DES.

Individual components	DES/base Complexes				
	**DES/Adenine**	**DES/Guanine**	**DES/Cytosine**	**DES/Thymine**	**DES/Uracil**
**Trehalose**	–0.175	–0.101	–0.039	0.035	–0.032
**Choline**	0.795	0.773	0.778	0.798	0.792
**Chloride**	–0.735	–0.794	–0.738	–0.755	–0.723
**Base**	0.115	0.054	–0.001	–0.077	–0.037
**Total charge**	0.000	0.000	0.000	0.000	0.000

Table S2. The electronic charges calculated for the DES and Base complexes using CHELPG method after DES interacted with the model nitrogenous bases.

Free Bases	
**Adenine**	0.000
**Guanine**	0.000
**Cytosine**	0.000
**Thymine**	0.000
**Uracil**	0.000

Table S3. The electronic charges calculated for the bases using CHELPG method prior to interaction with DES.

Free DES	
**trehalose**	–0.068
**choline**	0.829
**chloride**	–0.761
**Total**	0.000

Table S4. The electronic charges calculated for the DES using CHELPG method prior to interaction with bases DES

```
%mem=256GB

%nprocshared=32

# opt freq 6-31++g(d,p) m062x nosymm

DES and Adenine

0 1
 C                 -0.24011700    2.64599600    1.81761900
 C                 -1.25507100    2.68446900    0.66085600
 C                  0.44008300    3.11534900   -0.96566200
 C                  1.47259100    3.31807600    0.14002400
 C                  1.17467200    2.40713100    1.32061500
 H                 -1.43743400    1.64425000    0.34343300
 H                 -0.24361400    3.61521300    2.34069000
 H                  0.62828800    3.80015100   -1.79999900
 H                  1.36906800    4.36444700    0.47013600
 H                  1.26547600    1.36681500    0.97627700
 O                 -0.82361800    3.45485200   -0.45471800
 O                  0.40351100    1.78371500   -1.44755800
 O                  2.76637900    3.08008500   -0.34773300
 H                  3.34235900    3.07429100    0.43112600
 O                  2.14019300    2.68476000    2.30484200
 H                  2.38109800    1.84200300    2.73391100
 O                 -0.58870800    1.60314100    2.71907800
 H                 -1.50945300    1.76061300    2.98257300
 C                 -2.55813300    3.33280900    1.13093400
 H                 -3.22746700    3.50986000    0.28283000
 H                 -2.32752200    4.30792600    1.57193700
 O                 -3.18763200    2.54712800    2.13253500
 H                 -3.75952500    1.90636200    1.67714400
 C                  2.03618700   -1.12964100   -2.12831700
 C                  2.71394400    0.14403300   -1.61184600
 C                  1.07114500    1.51174400   -2.66244400
 C                  0.38795000    0.31307800   -3.33913300
 C                  0.56493200   -0.89570800   -2.43053400
 H                  2.34514700    0.36183000   -0.59842200
 H                  2.53575800   -1.43277300   -3.06372000
 H                  1.02543600    2.38827200   -3.31854600
 H                  0.93556800    0.12915700   -4.27073900
 H                  0.03291600   -0.70191000   -1.48160300
 O                  2.42962600    1.23105500   -2.48322500
 O                 -0.93709700    0.56019300   -3.70052900
 H                 -1.50850800    0.63829900   -2.91311300
 O                  0.01730100   -2.06579100   -3.04469200
```

H	0.55356200	-2.81294300	-2.73986100
O	2.06781700	-2.19807400	-1.19088800
H	3.00431600	-2.34705900	-0.96104600
C	4.24028800	0.00800200	-1.62055700
H	4.68246400	0.78989000	-0.99338000
H	4.57160400	0.16962300	-2.65167000
O	4.66539000	-1.28405600	-1.24233900
H	4.74065100	-1.36104800	-0.26946600
C	0.89413600	-1.15537600	1.91202000
H	0.15717200	-0.39331700	1.64260300
H	1.64837000	-1.26489600	1.12770100
C	-0.61930100	-2.60990700	0.67691900
H	0.09966600	-2.58448000	-0.14490500
H	-1.16034300	-3.55710100	0.70221900
H	-1.32690400	-1.78036400	0.60007100
C	1.09253500	-3.62765600	2.05081600
H	1.68553500	-3.54396900	2.95943100
H	0.50082400	-4.54521300	2.05642000
H	1.77028400	-3.58483700	1.19745700
C	-0.81191600	-2.47679000	3.09789500
H	-1.43940400	-1.58570800	3.02800400
H	-1.42453700	-3.37790900	3.03817200
H	-0.25092200	-2.46726500	4.03225000
C	1.56666700	-0.77976800	3.24014800
H	1.94839700	-1.67039100	3.75598400
H	0.85672700	-0.25896900	3.88871300
N	0.14385100	-2.47003100	1.95361900
O	2.62317400	0.10873800	2.98755500
H	3.34018800	-0.42274700	2.56079300
Cl	4.12121600	-2.13063100	1.65706000
N	-2.55741400	-2.20414300	-1.94457100
C	-3.07435000	-3.27310100	-1.25790500
H	-2.81215800	-4.28935600	-1.52043900
N	-3.89247500	-2.93850100	-0.28836100
C	-3.91393600	-1.56122400	-0.34345800
C	-4.56326000	-0.58073200	0.43450700
N	-5.32894700	-0.90438000	1.50072000
H	-5.91771100	-0.18663200	1.89659100
H	-5.60035800	-1.86894100	1.62021300
N	-4.38005200	0.71270000	0.12732300
C	-3.59289100	1.02019200	-0.92163900
H	-3.49817600	2.07577600	-1.15558800
N	-2.90597300	0.19721400	-1.69525800
C	-3.09450600	-1.08954700	-1.36853600
H	-1.79101700	-2.21075700	-2.63106100

```
%mem=256GB
%nprocshared=32
# opt freq 6-31++g(d,p) m062x nosymm

DES and Cytosine

0 1
 C                  2.36862000    2.80010900    0.67960400
 C                  3.60219200    2.11174400    0.06147300
 C                  3.78528100    0.58244900    1.85197200
 C                  2.71656100    1.30665400    2.65822700
 C                  1.68925000    1.88397000    1.69167800
 H                  3.25245800    1.33776500   -0.63640400
 H                  2.67832600    3.72061200    1.20094400
 H                  4.54915500    0.13266800    2.49529500
 H                  3.21316500    2.12908200    3.19621400
 H                  1.23490200    1.04027500    1.15640700
 O                  4.44560600    1.52597200    1.04441200
 O                  3.14720000   -0.39187700    1.07518500
 O                  2.11885800    0.40974500    3.55718500
 H                  1.31645000    0.84156400    3.88223700
 O                  0.70458100    2.55825800    2.44154200
 H                 -0.15473600    2.17440400    2.19682000
 O                  1.42022800    3.10532900   -0.33450700
 H                  1.87802900    3.65158300   -0.99013400
 C                  4.51313100    3.07776500   -0.67739800
 H                  5.33401900    2.50912200   -1.12798300
 H                  4.92477200    3.79723900    0.04225600
 O                  3.73956600    3.74597200   -1.67410100
 H                  4.28391100    4.40734100   -2.11262000
 C                  1.82845800   -2.82794500   -0.90128800
 C                  1.69936400   -2.70175400    0.62156400
 C                  3.80084900   -1.59615800    0.78555700
 C                  4.04343400   -1.70560400   -0.72206900
 C                  2.70274300   -1.70996100   -1.43130000
 H                  1.15301200   -1.77813300    0.86582200
 H                  2.30252500   -3.79750100   -1.13272400
 H                  4.74405200   -1.66878500    1.33809300
 H                  4.55053700   -2.66378800   -0.90922400
 H                  2.20301500   -0.74954500   -1.22130300
 O                  3.00386600   -2.67265600    1.20291600
 O                  4.84379100   -0.62494100   -1.14478900
 H                  4.92505500   -0.69210000   -2.10538800
 O                  2.93419100   -1.83114400   -2.82746000
 H                  2.19631900   -2.32440000   -3.21041400
 O                  0.58728700   -2.71530200   -1.58045800
```

H	-0.01200300	-3.35133800	-1.14535300
C	0.99481700	-3.91025800	1.23539800
H	0.80265500	-3.72181900	2.29718600
H	1.66259900	-4.77437500	1.15325100
O	-0.19472500	-4.23068200	0.53696700
H	-0.91357200	-3.63199400	0.82474200
C	-0.58532000	0.60122300	-0.87031300
H	0.32721000	1.20291600	-0.85217600
H	-0.37943200	-0.42686500	-0.55578300
C	0.19780300	0.27719400	-3.14808800
H	0.63789800	-0.66614800	-2.82589900
H	-0.10072500	0.21844200	-4.19634000
H	0.90067300	1.09891600	-2.99648800
C	-1.96783900	-0.62651000	-2.51887200
H	-2.87480600	-0.43396100	-1.94701200
H	-2.20837100	-0.67605200	-3.58320600
H	-1.46790400	-1.53474500	-2.18290800
C	-1.67552500	1.79382500	-2.74523700
H	-1.01447000	2.62351300	-2.48575900
H	-1.82753900	1.75247100	-3.82517600
H	-2.64235500	1.87821300	-2.24479500
C	-1.63084900	1.23428900	0.05305500
H	-2.63720200	0.87448800	-0.18048400
H	-1.62050400	2.32312700	-0.05384100
N	-1.01934100	0.51913200	-2.31927300
O	-1.28603500	0.95705100	1.39232100
H	-1.50304900	0.01621800	1.55944300
Cl	-2.25462500	-1.93141400	0.87926800
N	-4.99630400	-0.43359400	0.31762600
C	-5.80656800	-0.81839200	1.32600800
H	-5.40024300	-1.57632200	1.98802900
C	-7.03949800	-0.26472700	1.46293800
H	-7.71282200	-0.55610100	2.25745500
C	-7.38870500	0.72619000	0.48622500
N	-8.61076600	1.30985600	0.53998200
H	-8.80305900	2.05702000	-0.10912700
H	-9.23168100	1.15700900	1.31590400
N	-6.58934600	1.10977700	-0.49257600
C	-5.35575400	0.54782600	-0.61236600
O	-4.54119100	0.85874000	-1.48709700
H	-4.06780200	-0.86947400	0.25503800

```
%mem=256GB
%nprocshared=32
# opt freq 6-31++g(d,p) m062x nosymm

DES and Guanine

0 1
 C                 -0.77972100    2.60507100   -1.77769500
 C                 -2.29854000    2.46908300   -1.58278600
 C                 -2.28176800    0.19960600   -2.26843100
 C                 -0.81464400    0.27969400   -2.67418100
 C                 -0.09930200    1.24169300   -1.73851500
 H                 -2.49147900    2.21882900   -0.53343100
 H                 -0.56420000    3.06810900   -2.75477800
 H                 -2.84205200   -0.48318400   -2.91702500
 H                 -0.78876900    0.69264100   -3.69455700
 H                 -0.18491600    0.83529600   -0.71740500
 O                 -2.86583400    1.46888800   -2.42870100
 O                 -2.34558600   -0.21390500   -0.93195600
 O                 -0.23140200   -0.99794300   -2.64266400
 H                  0.72318000   -0.86811500   -2.76896500
 O                  1.24648700    1.28678700   -2.14459000
 H                  1.74836500    1.89757100   -1.56633300
 O                 -0.21412300    3.37654900   -0.72853300
 H                 -0.67068800    4.23055300   -0.72452900
 C                 -3.05745800    3.73300400   -1.95217600
 H                 -4.11783500    3.58817400   -1.71724300
 H                 -2.94668100    3.91033500   -3.03000900
 O                 -2.50766900    4.81563700   -1.20312200
 H                 -2.93915100    5.63660800   -1.45937500
 C                 -2.56932800   -1.71424400    2.06628200
 C                 -2.24782800   -2.40220500    0.73318000
 C                 -3.52960000   -0.81200900   -0.47461900
 C                 -3.97676800   -0.11235900    0.81154900
 C                 -2.85787400   -0.24599400    1.82737200
 H                 -1.30081300   -2.00774200    0.33419100
 H                 -3.47408700   -2.18527300    2.48972900
 H                 -4.31257100   -0.76528300   -1.23903300
 H                 -4.86714700   -0.64060300    1.18315700
 H                 -1.95549100    0.22782600    1.41002400
 O                 -3.30595900   -2.17216300   -0.19901800
 O                 -4.28169500    1.23594700    0.53748300
 H                 -4.43871300    1.66966500    1.38700000
 O                 -3.24316000    0.41899900    3.02070600
 H                 -2.77523400   -0.00599600    3.75278900
 O                 -1.50628000   -1.77338300    2.99841800
```

H	-1.13793400	-2.67541500	2.96027600
C	-2.13618500	-3.91055700	0.90792100
H	-1.98848500	-4.37559500	-0.07386700
H	-3.06696600	-4.30097600	1.33387000
O	-1.08345000	-4.22443900	1.80003100
H	-0.25474700	-4.14203200	1.27865300
C	1.49662000	1.78149500	1.39002300
H	0.45789500	1.90189400	1.07045200
H	1.91219800	0.87444800	0.94333900
C	0.39988200	0.52143900	3.16848800
H	0.57552200	-0.35910700	2.54980800
H	0.45354600	0.24857000	4.22429600
H	-0.57001400	0.96949200	2.94598900
C	2.78448000	0.98409800	3.35030900
H	3.59420800	1.63790800	3.02682100
H	2.74654200	0.90846600	4.43878200
H	2.90968700	0.00018300	2.89422000
C	1.14924700	2.79759600	3.60676000
H	0.23463600	3.21951300	3.18567700
H	1.00332200	2.56421900	4.66253700
H	1.97566800	3.49894600	3.49362500
C	2.31335000	3.00671000	0.95369100
H	3.17524000	3.16242000	1.61404600
H	1.68262200	3.89955400	0.96434300
N	1.46824400	1.53541400	2.88294100
O	2.76520800	2.82060800	-0.36532500
H	3.62885000	2.35545300	-0.27807800
Cl	5.09286500	1.23203500	0.73430100
N	1.26487600	-3.73410800	-2.44424600
C	0.52761200	-4.33258600	-1.45319400
H	-0.14105600	-5.15709300	-1.65526500
N	0.74898900	-3.80345700	-0.27944400
C	1.67028000	-2.80234600	-0.50952800
C	2.25365300	-1.83362000	0.36953900
O	2.03624800	-1.64947200	1.56794700
N	3.16815100	-1.02286900	-0.31164900
H	3.72688000	-0.35935000	0.25201200
C	3.40780800	-1.04108700	-1.66269700
N	4.29804600	-0.14660100	-2.11959000
H	4.82499800	0.43564600	-1.47324400
H	4.51318500	-0.16392400	-3.10263100
N	2.81290200	-1.87821900	-2.49752200
C	1.99783500	-2.74159400	-1.85740100
H	1.22250100	-3.92058600	-3.43517100

```
%mem=256GB
%nprocshared=32
# opt freq 6-31++g(d,p) m062x nosymm

DES and Thymine

0 1
 C                 -3.89858200    0.24859800    1.66551300
 C                 -3.36443100   -1.19056900    1.80661000
 C                 -3.82255600   -1.70158900   -0.45459800
 C                 -4.56670400   -0.39015100   -0.66099600
 C                 -3.91300800    0.68140100    0.20367000
 H                 -2.27811700   -1.17114300    1.64255300
 H                 -4.93000700    0.30302500    2.05053600
 H                 -4.24398300   -2.51074400   -1.06082600
 H                 -5.60310000   -0.54582300   -0.32265100
 H                 -2.87627200    0.79563500   -0.14095800
 O                 -3.97299100   -2.08968400    0.88960800
 O                 -2.47764000   -1.48473400   -0.77451900
 O                 -4.53098000   -0.04011500   -2.01889000
 H                 -4.79455200    0.88934000   -2.07178600
 O                 -4.61545600    1.88801700    0.01603600
 H                 -3.96199100    2.56258100   -0.23930000
 O                 -3.06096500    1.15130200    2.37716000
 H                 -3.02437400    0.83488800    3.29167000
 C                 -3.65582700   -1.80142200    3.16698000
 H                 -3.20245400   -2.79802300    3.20769800
 H                 -4.74298400   -1.89436800    3.28698500
 O                 -3.10853100   -0.94379800    4.16909300
 H                 -3.34895300   -1.27509600    5.03991600
 C                  0.84482200   -1.20848900   -1.47047700
 C                 -0.33260300   -0.94200400   -2.42131700
 C                 -1.71054200   -2.52772700   -1.31008300
 C                 -0.58981600   -2.89518200   -0.33526700
 C                  0.30585200   -1.67995100   -0.13187400
 H                 -0.92801500   -0.10336100   -2.03042100
 H                  1.48430000   -1.99525100   -1.90583000
 H                 -2.33988600   -3.39588600   -1.53495000
 H                 -0.00262400   -3.70413100   -0.79957700
 H                 -0.30661600   -0.87845000    0.30549600
 O                 -1.13895100   -2.11605700   -2.52277900
 O                 -1.14097500   -3.32015100    0.88857000
 H                 -0.41370800   -3.32533400    1.52682700
 O                  1.32223600   -1.96193100    0.81570100
 H                  2.03228000   -2.46196800    0.37171000
 O                  1.61677200   -0.03317300   -1.24398900
```

H	1.74491700	0.36548900	-2.13312500
C	0.13474700	-0.62545300	-3.84065000
H	-0.72794700	-0.33142500	-4.44815600
H	0.57223100	-1.53178800	-4.27303400
O	1.13815000	0.37422400	-3.84410900
H	0.71309000	1.25511100	-3.72294200
C	-0.58610400	2.23908700	0.52585600
H	-1.17755200	1.60464200	1.19305100
H	-0.32889200	1.70796300	-0.39589900
C	1.25522300	1.22129100	1.76668400
H	1.36759100	0.52616500	0.93619200
H	2.22827900	1.41012900	2.22317800
H	0.54128700	0.82995000	2.49436900
C	1.73044100	3.07497700	0.26299700
H	1.33501500	3.98868100	-0.17755200
H	2.66213600	3.24748000	0.80134600
H	1.87821400	2.33952000	-0.52658100
C	0.51874700	3.45845900	2.36117500
H	-0.27914700	3.06890100	2.99690500
H	1.45112000	3.53602900	2.92233600
H	0.24196100	4.43688700	1.96913900
C	-1.37808800	3.50811600	0.18278400
H	-0.71559400	4.29980100	-0.18909300
H	-1.91497000	3.87252700	1.06461600
N	0.72613700	2.51408500	1.22808100
O	-2.34599900	3.20258400	-0.78754200
H	-1.86134000	3.07986700	-1.63864700
Cl	-0.06595000	2.94944200	-2.76263800
N	5.76860100	-2.16174300	0.31763400
C	6.62054100	-1.20821800	0.83339600
H	7.61970600	-1.55841400	1.06938900
C	6.23562100	0.06695100	1.03547900
C	7.11772800	1.13679800	1.60050300
H	7.20901100	1.96899900	0.89713700
H	6.68830200	1.54175500	2.52108800
H	8.11426600	0.74634400	1.81763000
C	4.86014900	0.43168200	0.67892600
O	4.38178900	1.54346800	0.85048400
N	4.09474300	-0.58523000	0.11443200
C	4.46818400	-1.89487500	-0.04333000
O	3.71520100	-2.76815100	-0.46036200
H	6.06098200	-3.12200700	0.20319500
H	3.15713300	-0.34359800	-0.24539400

```
%mem=256GB
%nprocshared=32
# opt freq 6-31++g(d,p) m062x nosymm

DES and Uracil

0 1
 C                  2.43778400   -2.75824400   -0.72952800
 C                  3.65044200   -2.05559100   -0.08650700
 C                  3.81846500   -0.49022300   -1.84688800
 C                  2.77391300   -1.22187500   -2.67777600
 C                  1.74964900   -1.83916800   -1.73280900
 H                  3.27761400   -1.30214400    0.62199900
 H                  2.77121000   -3.66323300   -1.26289900
 H                  4.57882500   -0.01214300   -2.47361000
 H                  3.29353100   -2.02240100   -3.22670200
 H                  1.27210500   -1.01411300   -1.18949200
 O                  4.49047000   -1.43401600   -1.04964700
 O                  3.15102100    0.45570800   -1.05906600
 O                  2.16604400   -0.31931600   -3.56426300
 H                  1.39032600   -0.76933300   -3.92679000
 O                  0.78713000   -2.52298500   -2.50310600
 H                 -0.08061500   -2.14876500   -2.27716800
 O                  1.48657300   -3.10119900    0.27095500
 H                  1.95110700   -3.65164500    0.91856000
 C                  4.57377600   -3.01584300    0.64448300
 H                  5.37789500   -2.43880400    1.11405900
 H                  5.00761000   -3.71373500   -0.08321400
 O                  3.80353900   -3.71750700    1.62084200
 H                  4.35638700   -4.37662200    2.05221300
 C                  1.75747000    2.82006800    0.95061400
 C                  1.64861000    2.72418300   -0.57634400
 C                  3.77562600    1.66815100   -0.74022800
 C                  4.00145000    1.75409900    0.77137900
 C                  2.65400600    1.71335700    1.46709100
 H                  1.12464800    1.79451500   -0.84612300
 H                  2.20363700    3.79634000    1.20799700
 H                  4.72180800    1.77221000   -1.28235300
 H                  4.48498400    2.71953600    0.98175700
 H                  2.18047400    0.74559600    1.23233200
 O                  2.95871000    2.73491700   -1.14411500
 O                  4.82140300    0.68331400    1.18090300
 H                  4.90416800    0.74105200    2.14196200
 O                  2.86650600    1.80845700    2.86853200
 H                  2.13572500    2.31323600    3.24975900
 O                  0.51145600    2.66153600    1.61244300
```

H	-0.09834400	3.29403900	1.18718500
C	0.92678800	3.93157700	-1.17220900
H	0.74921300	3.76289800	-2.23972700
H	1.57682500	4.80618500	-1.06474100
O	-0.27517900	4.21567700	-0.47878800
H	-0.98247600	3.61461400	-0.78468400
C	-0.54291200	-0.64289600	0.83405600
H	0.39017100	-1.21080200	0.79898300
H	-0.37534500	0.40230700	0.55625900
C	0.22428300	-0.36345600	3.12553500
H	0.62533300	0.60781400	2.83777300
H	-0.07834600	-0.35425900	4.17424500
H	0.96104200	-1.14932900	2.94703100
C	-1.97569500	0.47002900	2.51917500
H	-2.86708800	0.26999100	1.92668300
H	-2.22790400	0.46975000	3.58188100
H	-1.51111800	1.41004300	2.22157200
C	-1.58071900	-1.94253500	2.66406500
H	-0.88484300	-2.73391000	2.37765500
H	-1.73910800	-1.94667400	3.74378900
H	-2.53875500	-2.05350800	2.15347000
C	-1.56372400	-1.28412200	-0.11149700
H	-2.58364900	-0.96928300	0.13152800
H	-1.51037600	-2.37487500	-0.04136300
N	-0.98081000	-0.62758400	2.28430500
O	-1.23220200	-0.94995200	-1.43979600
H	-1.48210700	-0.01271300	-1.57552800
Cl	-2.30991800	1.87355000	-0.78768100
N	-5.03935400	0.38203900	-0.34880600
C	-5.91362800	0.84021200	-1.30153500
H	-5.50981400	1.62634200	-1.93068900
C	-7.16205400	0.34772100	-1.43401900
H	-7.84978900	0.70925000	-2.18464900
C	-7.60956600	-0.71183400	-0.54076100
O	-8.69980200	-1.24910500	-0.53905400
N	-6.63156200	-1.11034600	0.39214200
C	-5.35699900	-0.61653700	0.53298600
O	-4.57441400	-1.04218700	1.38047200
H	-4.09988400	0.80474200	-0.30996500
H	-6.89621800	-1.85044200	1.03190400

```
%mem=256GB
%nprocshared=32
# opt freq 6-31++g(d,p) m062x nosymm

DES and Guanine-Cystocine Base Pair

0 1
 C                 -0.94594900   -1.16816600    2.80184100
 C                 -0.41248900   -2.19545100    1.78708300
 C                 -2.58544700   -2.70314400    0.98845500
 C                 -3.23886600   -1.85255000    2.07009600
 C                 -2.33896700   -0.67833200    2.42989400
 H                 -0.12505600   -1.66799200    0.86276400
 H                 -1.01611200   -1.64547400    3.79256900
 H                 -3.20810300   -3.56931100    0.73705400
 H                 -3.35527100   -2.49232000    2.95891300
 H                 -2.26481900   -0.03757100    1.53867400
 O                 -1.37246500   -3.20600400    1.48869900
 O                 -2.31883200   -1.94168300   -0.16390600
 O                 -4.48748800   -1.39357100    1.61729000
 H                 -4.78649500   -0.74508300    2.27132800
 O                 -2.96684800    0.00535800    3.48781000
 H                 -2.89699100    0.96915500    3.34394500
 O                 -0.07752600   -0.04351600    2.86128500
 H                  0.82292100   -0.37981100    2.99210400
 C                  0.79407900   -2.94224800    2.33557200
 H                  1.07900900   -3.72676200    1.63464000
 H                  0.52450200   -3.41207100    3.29117500
 O                  1.85005900   -2.00536900    2.52523800
 H                  2.71036900   -2.42133900    2.37654800
 C                 -2.97372400    0.50496100   -2.34178700
 C                 -3.96848700    0.05023200   -1.26422300
 C                 -3.07347900   -2.15192600   -1.32788100
 C                 -2.16734800   -1.82757800   -2.52548800
 C                 -1.73227000   -0.37513600   -2.35823400
 H                 -3.54963000    0.28395400   -0.27426900
 H                 -3.46690700    0.42830300   -3.32642500
 H                 -3.42546100   -3.18913700   -1.37744100
 H                 -2.78247600   -1.91336200   -3.43206300
 H                 -1.20535800   -0.27778900   -1.39590900
 O                 -4.21780400   -1.34754300   -1.37236900
 O                 -1.10561600   -2.74129000   -2.60308300
 H                 -0.25335600   -2.25568900   -2.57020100
 O                 -0.84428100    0.03014700   -3.40093300
 H                 -1.12729300    0.91037000   -3.68702700
 O                 -2.51877600    1.83367400   -2.13254900
```

H	-3.32082300	2.38720000	-2.07124700
C	-5.33522100	0.71716400	-1.44665400
H	-5.93131800	0.58082300	-0.53750900
H	-5.84202300	0.20137600	-2.26932400
O	-5.22435400	2.07740300	-1.80965600
H	-5.12242100	2.64317000	-1.01481100
C	-1.16742800	2.42387800	1.10494500
H	-0.72789000	1.47390500	1.41722200
H	-2.01172000	2.26357000	0.42788800
C	0.42729900	2.14878400	-0.70425500
H	-0.40403500	1.82383600	-1.33165200
H	1.20551300	2.64305300	-1.28620100
H	0.85597300	1.30552200	-0.15818500
C	-0.69550700	4.28592000	-0.45755100
H	-1.14519200	4.98705400	0.24392200
H	0.10730900	4.75884000	-1.02674200
H	-1.47793400	3.90116600	-1.11195400
C	1.00922000	3.57988700	1.16468700
H	1.34322500	2.72773200	1.76046100
H	1.82226700	3.93380300	0.52786400
H	0.65551400	4.37997800	1.81553600
C	-1.63798200	3.19755100	2.33966900
H	-1.77321500	4.26375800	2.11884700
H	-0.92383200	3.08560800	3.16150000
N	-0.10931800	3.12838800	0.29002900
O	-2.85526900	2.64292200	2.77293700
H	-3.53698900	2.95079800	2.12790200
Cl	-4.08119300	3.93301500	0.32825900
N	0.98957900	-4.22303600	-1.18612000
C	1.68677400	-5.08766900	-0.36821700
H	1.34993500	-6.10045100	-0.19943200
N	2.75104900	-4.54652500	0.16215400
C	2.76020500	-3.25888000	-0.33372600
C	3.58351400	-2.13303700	-0.02820900
O	4.48458800	-2.05389900	0.81689500
N	3.24317600	-1.00970500	-0.79081600
H	3.80092100	-0.16159500	-0.59996800
C	2.20061900	-0.93719600	-1.67859800
N	2.04513500	0.20309600	-2.37618600
H	2.59024400	1.03320100	-2.15668900
H	1.19123600	0.26368200	-2.92564700
N	1.36494300	-1.93550000	-1.87680200
C	1.67679500	-3.04874900	-1.17854000
N	5.49534000	3.52842200	-0.77830800
C	6.69522600	3.42597400	-0.14473100
H	7.33882900	4.29733100	-0.18223300

```
 C                  7.03433500    2.28229100    0.49078000
 H                  7.98115800    2.17926500    1.00313700
 C                  6.07708600    1.20248300    0.44477200
 N                  6.34069600    0.04239300    1.03952100
 H                  5.65964200   -0.73240800    0.99265100
 H                  7.22007200   -0.10035500    1.50950700
 N                  4.90543000    1.32650000   -0.18583700
 C                  4.57456300    2.47727500   -0.81588400
 O                  3.50744200    2.65073000   -1.41521700
 H                  0.08587600   -4.34839900   -1.63446200
 H                  5.23235100    4.36409100   -1.28305600
```

```
%mem=256GB
%nprocshared=32
# opt freq 6-31++g(d,p) m062x nosymm

DES and Thymine-Adenine Base Pair

0 1
 C                  3.44156100   -0.63555300   -2.69042400
 C                  4.06223800   -1.60314400   -1.66175900
 C                  5.25576100    0.12911800   -0.59030100
 C                  4.88634800    1.13555200   -1.67133300
 C                  3.48951400    0.80105400   -2.18111800
 H                  3.35962200   -1.71349700   -0.82293000
 H                  4.00684600   -0.68492600   -3.63524800
 H                  6.24667700    0.32740100   -0.16840000
 H                  5.61332500    1.02054800   -2.49017000
 H                  2.80520700    0.90336400   -1.33047400
 O                  5.31779100   -1.14782100   -1.17850100
 O                  4.26816600    0.18121800    0.40040600
 O                  4.93447800    2.43175500   -1.13629000
 H                  4.50035100    3.01265300   -1.77633000
 O                  3.14177200    1.72958900   -3.18371500
 H                  2.31391200    2.15483000   -2.90516200
 O                  2.07845000   -0.97646700   -2.91821200
 H                  2.07075600   -1.90259700   -3.20254500
 C                  4.36520700   -2.97304400   -2.24546000
 H                  4.75797800   -3.61434700   -1.44891900
 H                  5.12591700   -2.86364800   -3.02905700
 O                  3.15497300   -3.50482700   -2.78635500
 H                  3.34247300   -4.33568700   -3.23435700
 C                  2.00735600    0.16095300    2.95265600
 C                  2.79393500    1.36701900    2.42419400
```

C	4.61170700	-0.01400000	1.74517200
C	3.92881100	-1.27678600	2.27611300
C	2.42607000	-1.08749700	2.20215300
H	2.50255300	1.56312700	1.38105200
H	2.24135100	0.03469000	4.02403100
H	5.69892100	-0.08191200	1.86071900
H	4.22437900	-1.39729400	3.32879100
H	2.15283900	-0.96098200	1.14173200
O	4.19335300	1.08912700	2.50166300
O	4.34234300	-2.38565200	1.50924800
H	3.86388200	-3.15517900	1.84454300
O	1.79745200	-2.25484200	2.71474400
H	0.99381500	-1.98122200	3.17739700
O	0.60564200	0.27766500	2.76689200
H	0.34914000	1.13555900	3.15424700
C	2.56891800	2.61183400	3.28119900
H	3.02739400	3.47944200	2.79417100
H	3.06501000	2.45581400	4.24504700
O	1.19519700	2.82278700	3.54490300
H	0.77344200	3.24203700	2.76715000
C	0.19013900	0.40181500	-0.71715300
H	1.02508500	-0.22076600	-1.04641100
H	0.40671800	0.87753000	0.24456700
C	-0.48652600	-1.69518000	0.33905900
H	-0.12063800	-1.29983400	1.28603000
H	-1.32846700	-2.37332300	0.49971100
H	0.31442600	-2.20108500	-0.20468300
C	-2.04331900	0.16245500	0.28996100
H	-2.40781600	0.98843400	-0.31880100
H	-2.85013500	-0.54333100	0.49575900
H	-1.61233600	0.54844600	1.21579600
C	-1.49912700	-1.05159300	-1.77783300
H	-0.65284600	-1.39553300	-2.37662800
H	-2.18556300	-1.87516900	-1.57478500
H	-2.02578300	-0.24523200	-2.29058900
C	-0.09994000	1.46567500	-1.78299100
H	-1.09386200	1.91207600	-1.64207000
H	-0.04496000	1.02280400	-2.78246700
N	-0.96858700	-0.54032300	-0.47781200
O	0.89298500	2.46032400	-1.72714000
H	0.72848400	2.99295700	-0.91814200
Cl	-0.33234900	3.43019700	0.91578800
N	-3.05478300	-4.42855200	1.32383800
C	-2.80530500	-4.79649300	0.02186600
H	-2.18554600	-5.64963300	-0.21701900
N	-3.38359100	-4.01660700	-0.85444600

```
 C                  -4.06249500   -3.09187100   -0.08365400
 C                  -4.85655200   -1.97101400   -0.42104900
 N                  -5.09288500   -1.61610000   -1.69381100
 H                  -5.53621900   -0.72400900   -1.91256900
 H                  -4.75331400   -2.20343800   -2.43950600
 N                  -5.34307900   -1.22529900    0.58453600
 C                  -5.05615000   -1.55953500    1.85724500
 H                  -5.45241600   -0.88697600    2.61194800
 N                  -4.33511200   -2.58753200    2.29233300
 C                  -3.86620300   -3.32070000    1.27603100
 N                  -3.47111700    3.13618300    0.63774700
 C                  -3.29030700    3.50663400   -0.67099700
 H                  -2.52140400    4.25937800   -0.81656900
 C                  -3.98119400    2.95439600   -1.69640900
 C                  -3.79087400    3.32786400   -3.13522900
 H                  -3.02142300    4.09652400   -3.23554100
 H                  -3.49654400    2.45499700   -3.72631400
 H                  -4.72299100    3.70275700   -3.56767300
 C                  -4.91773800    1.88233900   -1.37640000
 O                  -5.52923200    1.22149300   -2.21627000
 N                  -5.06843100    1.60224600   -0.02639500
 H                  -5.57981800    0.74510500    0.21037300
 C                  -4.30020500    2.10224100    1.01212200
 O                  -4.34342500    1.64063200    2.13867600
 H                  -2.72846400   -4.88511400    2.16357700
 H                  -2.74626800    3.40772300    1.30205000


%mem=256GB
%nprocshared=32
# opt freq 6-31++g(d,p) m062x nosymm

DES and Uracil-Adenine Base Pair

0 1
 N                  -5.89668200   -3.06762500   -0.57883200
 C                  -7.05863800   -2.38160300   -0.84274600
 H                  -7.85288300   -2.96361000   -1.29572000
 C                  -7.17958200   -1.07655700   -0.54705200
 H                  -8.08436800   -0.51935800   -0.74234200
 C                  -6.05755100   -0.37134800    0.06977700
 O                  -6.09131500    0.79375800    0.41966000
 N                  -4.90733200   -1.14715500    0.25857500
 H                  -4.06182300   -0.66562000    0.61268200
 C                  -4.77568000   -2.48047700   -0.01769400
```

O	-3.76704100	-3.14150700	0.20255600
N	0.02175600	3.70414700	-0.75612500
C	-0.43762100	4.61408600	0.16119700
H	0.23412900	5.30325700	0.65460800
N	-1.72782500	4.53895800	0.37454100
C	-2.14258200	3.52332600	-0.46345800
C	-3.39443200	2.90468100	-0.66957300
N	-4.49467700	3.26338400	0.02332700
H	-5.23354900	2.56943600	0.07040500
H	-4.34204800	3.83237400	0.84313500
N	-3.49029800	1.94243400	-1.60179800
C	-2.38245200	1.56834800	-2.24884700
H	-2.51246600	0.78294800	-2.98925100
N	-1.13454600	2.01227100	-2.09211100
C	-1.07217300	2.99621100	-1.17875000
H	0.97323900	3.50809200	-1.05558300
H	-5.80527700	-4.05116500	-0.78683900
C	3.21764300	-1.22070400	-2.44606700
C	4.35798700	-0.84932900	-1.47949600
C	3.68513800	1.41075200	-1.39633200
C	2.66444700	1.21658900	-2.51254400
C	2.08989500	-0.19256600	-2.39788400
H	4.02031400	-1.05706100	-0.45491400
H	3.60058100	-1.25487500	-3.47906500
H	4.12528200	2.41479600	-1.41514200
H	3.20011100	1.31033000	-3.46920600
H	1.58505200	-0.26665500	-1.42468100
O	4.74553400	0.51238400	-1.59167300
O	3.02323600	1.18299400	-0.17886200
O	1.67559400	2.20451600	-2.41107000
H	0.80179500	1.84850800	-2.67608100
O	1.16622200	-0.40391600	-3.43556900
H	0.30774900	-0.59169400	-3.01843300
O	2.67210400	-2.48384400	-2.08533700
H	3.40719400	-3.11415700	-2.08900100
C	5.64067300	-1.61826300	-1.74865400
H	6.38251500	-1.33502300	-0.99373500
H	6.01502300	-1.34357000	-2.74327400
O	5.34773300	-3.01446400	-1.68674900
H	6.13182900	-3.51675800	-1.92933800
C	1.91126800	0.47407500	2.98671500
C	1.31867900	1.50383800	2.01682500
C	3.44940300	1.84207300	0.98360300
C	4.12263300	0.85830000	1.94583300
C	3.11232900	-0.19542800	2.35647700
H	0.90648700	0.98978900	1.13531600

H	2.23488100	1.00173400	3.90065800
H	4.13350600	2.66069600	0.73106900
H	4.42369800	1.42604700	2.83916800
H	2.78102700	-0.73585100	1.45380100
O	2.34007400	2.42627600	1.61667100
O	5.24648200	0.28187700	1.32216700
H	5.59682200	-0.37986400	1.93343800
O	3.74851400	-1.09919200	3.24795700
H	3.07882400	-1.43245800	3.86008500
O	0.99829200	-0.55941600	3.32136700
H	0.18821800	-0.09427300	3.61226600
C	0.23211900	2.33862200	2.69690700
H	-0.29162600	2.94803600	1.95591100
H	0.71494700	3.00406100	3.42128700
O	-0.68366400	1.52307100	3.40994000
H	-1.34309500	1.19178900	2.76433500
C	0.25212900	-2.19927500	0.08119000
H	1.22913600	-2.31207100	-0.39793200
H	0.21402900	-1.28362700	0.67935400
C	1.38265100	-3.46508100	1.82656300
H	1.53872200	-2.52569300	2.35784000
H	1.28822400	-4.29088200	2.53460000
H	2.19738200	-3.65325600	1.12471200
C	-1.01126800	-3.06606800	2.02826500
H	-1.94579400	-2.94155600	1.47938600
H	-1.07319500	-3.91238700	2.71620900
H	-0.76967800	-2.14356700	2.55550400
C	-0.16460200	-4.61735900	0.33494200
H	0.59672800	-4.74492400	-0.43747900
H	-0.12308400	-5.44038800	1.05030100
H	-1.15895500	-4.56532800	-0.10990300
C	-0.85267300	-2.16980600	-0.97854700
H	-1.83503200	-2.35626800	-0.53375400
H	-0.65715400	-2.91975300	-1.75186500
N	0.10482300	-3.34402500	1.06269500
O	-0.83258800	-0.92062200	-1.62370700
H	-1.18797000	-0.25213500	-1.00345100
Cl	-2.06563600	0.12103900	1.06448700

```
%mem=256GB
%nprocshared=32
# opt freq 6-31++g(d,p) m062x nosymm

DES (Trehalose and Choline Cloride)

0 1
 C                  2.71755300    1.38710500    0.62587300
 C                  3.06240600    0.25150300   -0.35987800
 C                  2.48016300   -1.42377000    1.19857500
 C                  2.32339000   -0.40919200    2.32262000
 C                  1.78827400    0.88816100    1.72682200
 H                  2.17958600    0.05644200   -0.98582700
 H                  3.63990500    1.76232100    1.09826800
 H                  2.84323500   -2.38945300    1.56591800
 H                  3.32313500   -0.23197500    2.74859200
 H                  0.80754400    0.66012500    1.29213000
 O                  3.45311900   -0.94285000    0.30239400
 O                  1.23756800   -1.56012200    0.56934800
 O                  1.44859400   -0.92216200    3.29214100
 H                  1.20984000   -0.18495300    3.87095100
 O                  1.64176700    1.83487000    2.76094400
 H                  0.70379600    2.09009400    2.78421900
 O                  2.05788100    2.44366000   -0.06376700
 H                  2.65180100    2.71678400   -0.77870300
 C                  4.24657600    0.58276600   -1.25282000
 H                  4.39813000   -0.24406000   -1.95518300
 H                  5.14159600    0.69387400   -0.62729900
 O                  3.95888900    1.79959800   -1.94341200
 H                  4.72673000    2.06353700   -2.46001800
 C                 -1.69985000   -2.08422200   -1.08119400
 C                 -1.44242200   -2.24193600    0.42286400
 C                  0.84251300   -2.80143700    0.05275700
 C                  0.68308600   -2.69961000   -1.46637800
 C                 -0.41035700   -1.69434700   -1.77526700
 H                 -1.17128700   -1.26522700    0.85089400
 H                 -2.04647300   -3.05451700   -1.47798800
 H                  1.56704600   -3.57938600    0.31724300
 H                  0.37603900   -3.68901600   -1.83642700
 H                 -0.08873700   -0.71485600   -1.38461800
 O                 -0.38178000   -3.17918600    0.62078300
 O                  1.91168800   -2.31007100   -2.03804900
 H                  1.76298300   -2.21224700   -2.98781500
 O                 -0.56337400   -1.60764400   -3.18608200
 H                 -1.50542200   -1.51653600   -3.38129600
 O                 -2.64099800   -1.06661700   -1.38666600
```

H	-3.41599700	-1.24306300	-0.81732000
C	-2.65580500	-2.81902700	1.15151200
H	-2.48802700	-2.77138200	2.23310300
H	-2.75675600	-3.87115200	0.86379400
O	-3.85076900	-2.16222400	0.77730100
H	-3.91591700	-1.30389200	1.25287900
C	-1.11437200	1.88882400	-0.17564700
H	-0.05461600	1.71771300	-0.37981700
H	-1.64618800	0.94886000	-0.00241000
C	-1.22783800	1.64584400	-2.59940100
H	-1.59288100	0.63078600	-2.44742600
H	-1.63689400	2.06803200	-3.51948100
H	-0.13633200	1.66185700	-2.62364700
C	-3.19556200	2.41730400	-1.40654300
H	-3.55976200	2.95607800	-0.53325300
H	-3.57566900	2.86034100	-2.32936100
H	-3.48830900	1.37123500	-1.31100900
C	-1.22670100	3.87650500	-1.62758500
H	-0.13767800	3.88606500	-1.54787100
H	-1.54370200	4.22756300	-2.61093400
H	-1.66385700	4.50548000	-0.85259600
C	-1.26080800	2.80611600	1.04535700
H	-2.25197600	3.27662700	1.07294200
H	-0.48981400	3.58366500	1.02754400
N	-1.69774400	2.47376600	-1.44615000
O	-1.05520900	2.05304300	2.21249600
H	-1.86245800	1.49907700	2.33435600
Cl	-3.74228800	0.77958200	1.61829900
